# Factors associated with the frequency of physician visits among North Korean defectors residing in South Korea: a cross-sectional study

**DOI:** 10.1186/s12913-015-0736-0

**Published:** 2015-03-07

**Authors:** Bo-Ram Wang, Young Dae Kwon, Wootack Jeon, Jin-Won Noh

**Affiliations:** Catholic Institute for Healthcare Management, the Catholic University of Korea, 222 Banpo-daero, Seocho-gu Seoul, 137-701 Korea; Department of Humanities and Social Medicine, College of Medicine and Catholic Institute for Healthcare Management, the Catholic University of Korea, 222 Banpo-daero, Seocho-gu Seoul, 137-701 Korea; Department of Medical Education, Yonsei University College of Medicine, 50-1 Yonsei-ro, Seodaemun-gu Seoul, 120-752 Korea; Department of Healthcare Management, Eulji University, 212 Yangji-dong, Sujeong-gu, Seongnam, Gyeonggi-do 461-713 Korea

**Keywords:** North Korean defector, Physician visits, Health care utilization, Refugee medical support programs, Health status

## Abstract

**Background:**

Since the mid-1990s, a growing number of North Korean defectors have been arriving in South Korea, many of whom have various somatic and mental disorders. The health status of defectors is an important predictor of their successful resettlement. Therefore, this study examined the frequency of physician visits among North Korean defectors residing in South Korea, as well as the factors associated with this frequency.

**Methods:**

The data used in this study were collected through survey questionnaires and interviews conducted from April 6 to May 20, 2009, and involving 500 North Korean defectors who entered South Korea in 2007. This study used three domains of independent variables: ‘health-related factors,’ ‘special characteristics of North Korean defectors,’ and ‘demographic and socio-economic factors’. Nested multivariable linear regression analysis was conducted in order to determine the factors related to the frequency of physician visits between January 1 and December 31, 2008.

**Results:**

The average number of physician visits made by the participants during 2008 was 15.3; 14.5% of participants did not have physician visits. The number of physician visits was largely associated with health-related variables including disability, chronic disease and self-rated health status. The frequency of physician visits was higher among those with a disability, chronic disease, lower self-rated health score, a greater number of traumatic experiences during their escape, lower annual family income, and among females.

**Conclusions:**

This study confirmed that the number of defectors’ physician visits was related with objective and subjective health status, traumatic experiences during their migration, economic, and demographic variables. The results serve useful understanding of medical utilization characteristics among North Korean defectors in South Korea.

**Electronic supplementary material:**

The online version of this article (doi:10.1186/s12913-015-0736-0) contains supplementary material, which is available to authorized users.

## Background

North and South Korea separated due to variations in their politico-social environments during the Cold War. Since the 1980s, South Korea has experienced great improvement in its standard of living through rapid economic growth and modernization, whereas North Korea has suffered consecutive natural disasters and great economic hardship as a result of the collapse of the Communist bloc [1]. Since the mid-1990s, there has been a growing number of North Koreans escaping to China, Russia [1,2] and South Korea [1]. The cumulative number of North Korean defectors entering South Korea has grown from 10,000 in 2007 to 20,000 in 2010, and exceeded 24,000 in 2013 [3].

Newly arrived refugees, including North Korean defectors, tend to require urgent care for somatic and mental diseases [4-8]; thus, host countries examine refugees’ health status upon their arrival and offer various healthcare support services [5,8-11]. Immigrants and refugees in their initial settlement stage suffer numerous health problems, and therefore healthcare systems are critical in supporting social adaptation into their new society [12]. North Korean defectors residing in South Korea also experience various health problems when adapting to their new society and culture [7,13]; the South Korean Medical Aid Act covers them in order to lower economic barriers to medical services utilization [14].

Generally, refugees have difficulty in accessing healthcare services in host countries due to cultural and language differences [6,15]. Refugees’ health-seeking behaviors and attitudes, which are consistent with the norms or culture of their country of origin, often become barriers to medical services utilization in their host country [6]. North and South Koreans lived under a unified culture and language [16] but since the national division, the two countries have situated under the disparate ideology without mutual interchange. Thus, North Korean defectors may experience cultural and language gaps and experience lack of understanding of the South Korean health systems, variations in treatment-seeking behaviors, and difficulties in communication with medical staff [10].

Also, the financial burden of medical care is a significant hindering factor in their use of medical services [6]. Although most refugees are financially supported by host country governments, refugees often are unable to receive required treatments as they are restricted to those medical services or institutions covered by their healthcare support systems [17]. These barriers to medical services among refugees are similarly applicable to North Korean defectors living in South Korea. North Korean defectors experience the financial burden of medical care, such as co-payment, in spite of the Medical Aid Act because most are economically vulnerable [10,18].

The health status of North Korean defectors is a key predictor of their social adaptation level in South Korea [7]. Defectors with health problems face difficulties in the initial settlement process. Therefore, it is important to lower the barriers to utilization of healthcare services and to promote the health of defectors from the beginning of their resettlement. There are several studies related to health of North Korean defectors in South Korea; however, most focus on mental health and trauma experiences in North Korea and during their migration [19-21]. Some studies investigated the use of healthcare services among North Korean defectors, but most focused on current utilization status [18,22-25]; therefore no previous study has examined the factors associated with their healthcare services utilization. Considering that refugees enter host countries with various health problems, the early North Korean defectors in South Korea would likely have high physical health needs. Therefore, this study is aimed to investigate the frequency of physician visits and factors related to these visits among North Korean defectors living in South Korea.

## Methods

### Subjects and data source

This study used data from the ‘North Korean Defectors Panel’ in order to analyze their healthcare services utilization and relating factors. The data was collected by questionnaires (Additional file 1) and interviews with 500 individuals who agreed to participate in the panel study (from a total of 2,138 North Korean defectors aged 20–69 years), all of whom had entered South Korea between January and December 2007. Although this study attempted to analyze the entire population of defectors who entered the country in 2007, 1,447 individuals of the original total were unable to be contacted, and 107 individuals declined to participate. Another 84 individuals either abandoned the study or were unavailable for the survey. Two participants were excluded due to insufficient data, resulting in a total of 498 participants included in this study. The survey was conducted from April 6 to May 20, 2009. The survey was comprised of quantitative and qualitative observation in compliance with qualitative research review guidelines - RATS [26]. The study procedures and required written consent were verified and approved by the Institutional Review Board (IRB) of Severance Hospital prior to the start of the interviews. Informed written consent was obtained from all study participants according to the IRB-approved procedures.

### Variables

The dependent variable of this study was the frequency of physician visits: the number of times each subject received outpatient care between January 1 and December 31, 2008. The physician visits included outpatient services received in private clinics, urgent care, government clinics, excluding inpatient care. A natural logarithmic transformation was performed on the dependent variable to adjust right-skewed distribution, which is typical of medical utilization data [27]. By adding ‘one’ to the frequency of physician visits for every subject before the natural logarithmic transformation, this study took into account those subjects who had not made physician visits.

This study set three domains of independent variables in order to determine the factors associated with frequency of physician visits. The domains were ‘health-related factors’, ‘special characteristics of North Korean defectors’, and ‘demographic and socio-economic factors’. Health-related factors included disability status, chronic diseases, and self-rated health status. Both disability status and chronic diseases were measured through binary yes-no questions. Self-rated health status was assessed using a Likert five-point scale [28], with a higher score indicating better health.

Special characteristics factors of North Korean defectors included exile duration, residence period in South Korea, level of satisfaction with South Korean government support, satisfaction with current life, language barrier, cultural barrier, traumatic experiences in North Korea, and traumatic experiences during migration. The exile duration was defined as the period from exodus date to December 31, 2008, which included time spent in other countries as well as South Korea; it was classified as less than 5 years, 5–10 years, or 10 years or longer. Residence period in South Korea was defined as the period from their entrance date to December 31, 2008; a median split was used to classify the residence period: less than 18 months vs. 18 months or longer. A five-point scale was used in order to assess the level of satisfaction with South Korean government support and current life satisfaction, with a higher score indicating greater satisfaction. Language and cultural barriers were defined as the level of difficulties experienced overall with regard to navigating South Korean language and culture were measured using a five-point scale, with a higher score representing greater difficulties. Traumatic experiences were measured using questionnaires developed by Kang [29], which included 17 questions for assessing traumatic experiences in North Korea and 18 questions for measuring traumatic experiences during migration, with one point allocated for each question. A higher score on the traumatic experiences scale signified more traumatic events.

Demographic and socio-economic factors consisted of gender, age, marital status, education level, religion, employment status, annual household income, and health insurance type. All variables were obtained from December 31, 2008 except education level, which was measured based on the level of education completed in North Korea. Marital status was categorized as single, married, or unmarried, which included divorced, separated, and widowed. The level of education completed in North Korea was classified as secondary school graduate or less or some college or higher. Annual household income included earned income and government aid in 2008. Natural log transformation was applied to annual household income in order to correct for its skewed distribution. Health insurance type was classified as Medical Aid and National Health Insurance (NHI). Republic of Korea has a compulsory NHI system with universal coverage and the system covers the majority of the population, excluding a small portion who are covered by Medical Aid program (an alternative welfare program for poor) [30].

### Statistical analysis

We performed a descriptive analysis and univariable analysis, including t-test, one-way ANOVA, and simple linear regression in order to identify differences in the frequency of physician visits according to the independent variables (health-related factors, special characteristics of North Korean defectors, and socio-economic and demographic characteristics). Every independent variable was used in the multivariable analysis. Nested linear models were applied to determine factors associated with the frequency of physician visits among North Korean defectors. The first model included health-related factors; the second model added special characteristics of North Korean defectors; and the third model added demographic and socio-economic factors. Data was analyzed using the Statistical Package for the Social Sciences, version 18.0 (SPSS Inc., Chicago, IL, USA).

## Results

### The frequency of physician visits according to general characteristics

Seventy-two participants (14.5% of the total) did not have physician visits between January 1 and December 31, 2008. The average frequency of physician visits for all study participants was 15.3 (±27.3); the median was 6. The percentage of respondents who made physician visits fewer than 10 times and fewer than 30 times was 66.3% and 89.0% respectively.

Table 1 illustrates the frequency of physician visits according to participants’ general characteristics. The natural logarithm was used to compensate for skewed distribution and variation in physician visit frequency across the study participants. Participants with disabilities (2.80) or chronic diseases (2.77) had more physician visits than those without disabilities (1.91) or chronic diseases (1.38). Individuals with a higher self-rated health score made fewer physician visits. The frequency of physician visits increased among those who had traumatic experiences in North Korea. Female subjects (2.11) made more physician visits than males (1.43), and the number of visits increased with age. The highest frequency (2.21) was found in unmarried participants, while the lowest frequency (1.75) was found in single subjects. Employed subjects (1.74) made fewer physician visits than the unemployed (2.23), and visiting frequency declined with annual household income growth. The Medical Aid beneficiary group (2.09) had a greater number of physician visits than those with NHI (1.59) (Table 1).

**Table 1:** **Relationships between general characteristics and frequency of physician visits**

		**N (%)**	**Mean ± S.D/beta**	**t/F**	***p*** **-value**
Frequency of physician visits^*^			1.97 ± 1.28		
Disability	yes	36 (7.2)	2.80 ± 1.16	−4.08	<.001
	no	462 (92.8)	1.91 ± 1.27		
Chronic disease	yes	214 (43.0)	2.77 ± 1.16	−14.02	<.001
	no	284 (57.0)	1.38 ± 1.01		
Self-rated health score			2.78 ± 1.14/-.523	−13.67	<.001
Exile duration	<5 years	256 (51.4)	1.94 ± 1.24	2.98	0.052
	5 ≤ ~ <10 years	135 (27.1)	2.20 ± 1.28		
	≥10 years	104 (20.9)	1.81 ± 1.34		
Residence period in SK	<18 months	243 (48.8)	1.92 ± 1.20	−1.03	0.304
	≥18 months	253 (50.8)	2.04 ± 1.35		
Satisfaction with SK government support			3.78 ± 1.00/-.015	−0.34	0.737
Life satisfaction			3.22 ± .89/-.023	−0.522	0.602
Language barrier			2.32 ± .99/.028	0.388	0.533
Cultural barrier			2.10 ± .74/.007	0.154	0.877
Traumatic experiences in NK			11.93 ± 3.18/-.108	−2.43	0.015
Traumatic experiences during migration			15.05 ± 2.73/-.024	−0.54	0.591
Gender	male	101 (20.3)	1.43 ± 1.26	−4.96	<.001
	female	397 (79,7)	2.11 ± 1.25		
Age	20-29	128 (25.7)	1.67 ± 1.08	7.05	0.001
	30-39	214 (43.0)	1.97 ± 1.32		
	≤40	156 (24.5)	2.23 ± 1.32		
Marital status	single	152 (30.5)	1.75 ± 1.28	5.03	0.007
	married	199 (40.0)	1.97 ± 1.22		
	unmarried^†^	146 (29.3)	2.21 ± 1.32		
Education in NK	secondary school graduate	375 (75.3)	2.01 ± 1.30	1.11	0.267
	some college or more	123 (24.7)	1.86 ± 1.21		
Religion	yes	237 (47.6)	2.05 ± 1.30	−1.17	0.244
	no	261 (52.4)	1.91 ± 1.26.		
Job	yes	252 (50.6)	1.74 ± 1.20	4.3	<.001
	no	220 (44.2)	2.23 ± 1.31		
Annual household income^‡^			16.46 ± .74/-.266	−5.24	<.001
Health insurance type	Medical Aid	370 (74.3)	2.09 ± 1.30	4.1	<.001
	NHI	120 (24.1)	1.59 ± 1.11		
Total		498 (100.0)			

*Frequency of physician visits was log-transformed.

†Unmarried included divorced, separated, and widowed.

‡Annual household income was log-transformed.

SK, South Korea; NK, North Korea; NHI, National Health Insurance.

### Factors associated with the frequency of physician visits

Table 2 presents the results of nested multivariable linear regression analysis, which sought to determine factors relating to the frequency of physician visits. In the first model, which includes only the health-related factors (Model 1), all three health-related variables were associated with the frequency of physician visits. The frequency of physician visits was significantly higher among those with disabilities, chronic diseases, or lower self-rated health scores. With the addition of the special characteristics for North Korean defectors to the model (Model 2), all three health-related variables remained significant factors, and two special characteristics of North Korean defectors (residence period in South Korea and traumatic experiences during migration) were statistically significant. The number of physician visits increased among those participants who had been living in South Korea longer than 18 months and had undergone more traumatic experiences. With the addition of demographic and socio-economic factors to the model (Model 3), all health-related factors, as well as traumatic experiences during migration, remained statistically significant. Two demographic and socio-economic factors, gender and annual household income, were associated with frequency of physician visits. The number of physician visits was higher among females, but decreased with annual family income growth. The fitness and the explanation level of the models were improved with the addition of independent variables. The final model (Model 3) is the best model for this analysis. Since the average values of the variation inflation factor in all three models were lower than 10, there was no multicollinearlity among the variables (Table 2).

**Table 2:** **Nested linear models for frequency of physician visits among North Korean defectors**

	**Model 1**		**Model 2**		**Model 3**	
	**beta**	***p*** **-value**	**beta**	***p*** **-value**	**beta**	***p*** **-value**
Disability (no)						
yes	.077	.036	.086	.022	.090	.045
Chronic disease (no)						
yes	.356	<.001	.347	<.001	.346	<.001
Self-rated health	-.297	<.001	-.326	<.001	-.287	<.001
Exile duration (<5 years)						
5 ≤ ~ <10 years			.069	.079	.051	.292
≥10 years			.005	.903	-.039	.446
Residence period in SK (<18 months)						
≥18 months			.082	.026	.032	.459
Satisfaction with SK government support			.024	.537	-.005	.911
Life satisfaction			.018	.650	.048	.320
Language barrier			-.042	.255	-.071	.111
Cultural barrier			-.065	.090	-.048	.290
Traumatic experiences in NK			-.037	.373	-.078	.126
Traumatic experiences during migration			.083	.048	.108	.033
Gender (male)						
female					.160	.001
Age (20–29)						
30-39					-.042	.478
≤40					-.053	.408
Marital status (single)						
married					.073	.179
unmarried^†^					.055	.343
Education in NK (secondary school graduate)						
some college or more					-.008	.858
Religion (no)						
yes					-.056	.201
Job (no)						
yes					.041	.429
Annual household income^‡^					-.110	.028
Health insurance type (Medical Aid)						
NHI					-.007	.884
F-value (*p*-value)	93.34 (<.001)		24.92 (<.001)		11.13 (<.001)	
−2 log-likelihood (*p*-value)	1655.643 (<.001)		1643.644 (<.001)		1145.889 (<.001)	
R^2^ (adj. R^2^)	.362 (.358)		.383 (.368)		.429 (.390)	
Mean VIF	1.385		1.251		1.449	
Number of observation	498		498		498	

†Unmarried included divorced, separated, and widowed.

‡Annual household income was log-transformed.

SK, South Korea; NK, North Korea; NHI, National Health Insurance; VIF, variance inflation factor.

## Discussion

This study evaluated the frequency of physician visits among 498 North Korean defectors who entered South Korea in 2007; it also analyzed the factors associated with this frequency. The average number of physician visits utilized during 2008 was 15.3, which is similar to the number of physician visits among the general South Korean population (15.4) during the same period [31]. Even though this study did not survey unmet medical needs, as North Korean defectors have relatively greater health needs than the general South Korean population [4,6,12,13], it may be assumed that their unmet medical needs would be more serious than those of South Koreans [19,32]. Therefore, further researches should focus on unmet medical needs and impediments of medical utilization of North Korean defectors residing in South Korea in order to improve their health.

Health-related variables had the greatest association with the frequency of physician visits among North Korean defectors. Previous studies have also found that health-related factors, such as perceived quality of general health and number of somatic symptoms, were significant factors affecting refugees’ use of healthcare services [15,33]. This study showed that the number of physician visits was higher among individuals with disabilities, chronic diseases, or lower self-rated health scores. It is generally known that physically disabled people have impeded mobility; therefore their access to healthcare services tends to be low [34,35]. This study data only specified whether or not participants had disabilities, and did not distinguish whether their level of disability interfered with their access to healthcare services. More research is needed to identify the relation of disability level on the utilization of healthcare services among North Korean defectors through assessment of the severity of their disabilities. Participants with chronic diseases and low self-rated health scores made more physician visits. Although this study did not investigate the onset timing of chronic diseases, the increased prevalence of chronic diseases among North Korean defectors during the resettlement process suggests that governmental support is essential to prevent such diseases during early resettlement [7]. The results showed that the frequency of physician visits increased with low self-rated health score. In general, unmet medical needs escalate alongside a lower self-rated health status [36]. Although there was no data available related to unmet medical needs, it is forecasted that unmet medical needs are likely more serious among North Korean defectors since their self-rated health status is remarkably low compared to that of South Koreans [7,15,33]. Future studies needs to focus on the unmet medical needs of North Korean defectors.

Previous studies on utilization of healthcare services among refugees have shown that their particular characteristics, such as language barriers and cultural differences, are obstacles to medical care [5,15,37]. However, this study presented different results: the frequency of physician visits by North Korean defectors was associated with their residence period in South Korea and traumatic experiences during migration, rather than cultural or language gaps. It is assumed that the differences in findings are caused by that North Korean defectors living in South Korea are different from refugees or immigrants in host countries with a completely different language or culture. North and South Korea were one nation before their division; therefore North Korean defectors likely experience relatively fewer language or cultural challenges, which may explain why these variables had no association with healthcare utilization. This study found that participants had undergone various traumatic experiences, both in North Korea and during their defection. According to Lawrence and Kearns, refugees are easily exposed to traumatic experiences, which can cause intense physiological distress [6]. Because the traumatic experiences of North Korean defectors may pose a threat to their physical and mental health, their general health status is poorer than that of the general population in South Korea [7,21]. The number of physician visits increased among participants who experienced more traumas during their defection, because a greater number of traumatic experiences are linked to exposure to unhealthy environments and risky behavior [22]. This may have led to an escalation in disease outbreak or disability among participants.

Female participants made more physician visits than males, and this trend is also observed among the general population [38,39]. The number of female North Korea defectors entering South Korea from the early 2000s was greater than that of males, and females comprised 78% of defectors entering the country in 2008 [40]. Since the collapse of the North Korean healthcare system in the 1990s, North Korean women were unable to receive proper care during pregnancy and childbirth, and their postpartum health deteriorated [41]. Female defectors are also frequently exposed to human trafficking and sexual abuse during their migration from North Korea, and thus many of them arrived in South Korea with obstetric disorders [39]. However, due to a lack of awareness with regard to prevention and management of such disorders, most female defectors do not receive screening exams or proper medical treatment [42]. Previous studies determined that many female refugees were unable to receive screening or medical treatment for diseases related to female health [5,43]. It is recommended that the South Korean government pay greater attention to female defectors and provide them with education in order to ensure proper utilization of healthcare services.

In general, low-income groups utilize healthcare services less frequently than high-income groups because financial burden prohibit their access of medical care [44,45]. Also the study regarding medical utilization of general South Korean population found same result as well [34]. In this study, however, the frequency of physician visits decreased with increasing annual household income. It is assumed that conflicting results are the result of income disparities between North Korean defectors and the general public of South Korea. The 2008 average annual household income of North Korean defectors was 8,780,000 Korean won (KRW) (approximately 7,803 USD), which is considerably low compared with the 30,790,000 KRW (approximately 27,365 USD) income of South Koreans surveyed between 2007 and 2009 [46]. Considering previous study results, which indicate that the unemployment rate of North Korean defectors reaches 3-40% and the beneficiary rate for the National Basic Livelihood Security Program is greater than 80% [47,48], socio-economic classification is not distinct among North Korean defectors [7]. Therefore, it is difficult to apply the perspective of equity in healthcare services according to the economic level of the general public to North Korean defectors.

Although insurance type had no significant relation to multivariable analysis, the Medical Aid beneficiary group made more physician visits than the NHI group. It is assumed that North Korean defectors with NHI had greater co-payment expenditures than Medical Aid beneficiaries. The ratio of North Korean defectors giving up medical treatment due to the burden of medical expense was 23.9%, more than twice that of South Koreans at 11.4% [7]. When North Korean defectors obtain employment in South Korea, they are mandatorily covered by the four major public insurance systems including workmen’s compensation, labor, health, and employment insurance; employed defectors are uniformly excluded from Medical Aid program, and thus their financial burden of medical expense is greater. Since the average income of North Korean defectors is only 57% of the average income of the general public in South Korea [2], some defectors intentionally avoid finding employment in order to receive Medical Aid benefits [18]. It is important that the South Korean government differentiates the scope and targeting of its healthcare coverage for North Korean defectors from that of South Koreans by considering their economic and health vulnerabilities.

The results of this study are not generalizable to all North Korean defectors living in South Korea, since random selection was not used for sampling. However, this limitation was inevitable because the total number of subject was very low and defectors tend to concern identity exposure and to avoid external contact. In addition, this study analyzed data collected through cross-sectional study; therefore, it cannot detect a causal relationship between independent variables and frequency of physician visits. Recall bias might have influenced the data collection of the frequency of physician visits.

## Conclusions

This study analyzed factors associated with frequency of physician visits among North Korean defectors residing in South Korea. This study confirmed that the number of defectors’ physician visits was related with objective and subjective health status, traumatic experiences during their migration, economic, and demographic variables. These findings help clarify the characteristics of medical utilization among North Korean defectors in South Korea.

## Additional file

Additional file 1:
**Questionnaire for demographic and economic characteristics among North Korean defectors.**

## Abbreviations

IRB: Institutional Review Board
NHI: National Health Insurance
ANOVA: Analysis of variance
KRW: Korean won
