# Supplementary material for: Factors associated with the frequency of physician visits among North Korean defectors residing in South Korea: a cross-sectional study
Source: BMC Health Serv Res. 2015 Mar 7;15:90. doi: 10.1186/s12913-015-0736-0 (PMC4377198; doi:10.1186/s12913-015-0736-0)
Supplement: Additional file 1: — Questionnaire for demographic and economic characteristics among North Korean defectors. [file 12913_2015_736_MOESM1_ESM.pdf]

이 조사표에 작성된 내용은 통계법 제13조에 의하여 비밀이 보장됩니다

북한이주민의 남한사회 적응 변화조사

A
(가족 면담용)

응답자 주소지	______[add1]______ 시 도 ______[add2]______ 구·시 군 ______[add3]______ 동·읍·면

[add] : 지역에 대한 구분 5개 광역시와 11개시도로 나눈 변수

1. 가족 사항 (a1)

본 조사 시점은 2008년 12월 31일입니다. 면담자의 안내에 따라 말씀해주시고, **응답자 본인을 1번에 기입**해 주시고 함께 사시는 분은 2번 부터 작성하십시오.

q01s0)귀하와 함께 사시는 분은 모두 몇 명입니까?
(※ 2008년 1년 동안 9개월 이상 생계를 같이한 사람을 기준으로 말씀하여 주십시오.
단, 직장 때문에 따로 사는 세대주, 학생, 기타 이유로 같이 살지 않는 가족도 포함하여 주십시오.)

	명

가족 성명	q01) 성별	q02)세대주와의 관계	q03)최종 탈북연월		q04)한국입국		q05) 태어난 연월		q06) 출 생 지	
순서대로 기입	① 남<br>② 여	※세대주 : 가구를 실질적으로 대표하고 생계를 책임지고 있는 사람	연도 [s1]	월 [s2]	연도 [s1]	월 [s2]	※주민등록 생년월		출생국가 [s1]	북한지역 [s2]
							년도 [s1]	생월 [s2]	① 북한→**문6-2)로 이동**<br>② 중국-<br>③ 남한→ **문7)로 이동**<br>④ 기타-<br><br>※ **기타는 직접 기록**	① 함경북도 ② 함경남도<br>③ 평안북도 ④ 평안남도<br>⑤ 황해북도 ⑥ 황해남도<br>⑦ 양강도 ⑧ 자강도<br>⑨ 평양 ⑩ 개성<br>⑪ 강원도

번호	q07) 교육 수준		q08) 북한취득 자 격 증	q09) 혼인 상태		q10) 종교
	북한 [s1]	남한 [s2]		[s1] 현재 혼인 상태	[s2] 배우자의 국적	
순서대로 기입	① 무학<br>(만 8세 이상)<br>② 인민중퇴<br>③ 인민졸업<br>④ 고등중 중퇴<br>⑤ 고등중 졸업<br>⑥ 전문대 중퇴<br>⑦ 전문대 졸업<br>⑧ 대학 중퇴<br>⑨ 대학 졸업	① 없음<br>② 초등 재학/졸업<br>③ 중등 재학/졸업<br>④ 고등 재학/중퇴<br>⑤ 고등 졸업<br>⑥ 대학 재학<br>⑦ 대학 중퇴<br>⑧ 대학 졸업<br>⑨ 고입검정합격<br>⑩ 대입검정합격<br>⑪ 대학원 재학/졸업	**직접 기록**	⓪ 비해당 → **문10)으로 이동**<br>(남17세 이하 여15세 이하)<br>① 미혼 → **문10)으로 이동**<br>(남18세 이상 여 16세 이상)<br>② 결혼 신고하고 동거<br>③ 결혼 신고하지 않고 동거<br>④ 이혼<br>⑤ 별거<br>⑥ 사별	① 북한 출신<br>② 남한 출신<br>③ 조선족<br>④ 중국 한족<br>⑤ 기타(**직접 기록**)	① 기독교<br>② 천주교<br>③ 불교<br>④ 없음<br>⑤ 기타(**직접 기록**)

2. 경제활동상태 (a2)

※ 만14세 이하인 경우는 문1)의 '0'으로 작성하고, [4. 건강]으로 이동합니다. 번호와 이름은 1쪽의 순서 및 내용과 일치하도록 작성하여 주십시오.

번호	q01) 근로 능력 정도	q02) 주된 경제활동 참여 상태	q03) 근로 무능력 이유	q04) 월평균근로/사업 소득액
	⓪ 만14세 이하→ **[4.건강]으로 이동**<br>① 근로가능 **문 2)로 이동**<br>② 단순근로 가능<br>③ 근로능력이 없어 경제활동하지 않음 → **문 3)으로 이동**	① 정규직 근로자<br>② 계약직 근로자 | **문 4)로 이동**<br>③ 일용직 근로자<br>④ 자활근로/공공근로<br>⑤ 고용주<br>⑥ 자영업자 **문 4)로 이동**<br>⑦ 무급가족종사자<br>⑧ 실업자(지난 4주간 적극적으로 구직) → **문 6)으로 이동**<br>⑨ 비경제활동인구 → **문 6)으로 이동**	① 심한 장애<br>② 질병 또는 부상<br>③ 노령으로 인한 무능력<br>④ 기타(**직접 기록**)	

[응답방법]

문 2) 주된 경제활동 참여 상태

① 정규직 근로자 : 고용 계약기간이 1년 이상인 사람 또는 특별한 고용계약이 없어 기간이 정해져 있지 않더라도 계속 정규직원으로 일하면서 상여금, 수당 및 퇴직금 등을 받는 사람
② 계약직 근로자 : 고용 계약 기간이 1개월 이상 1년 미만인 사람 또는 일정한 사업 완료의 필요성에 의해서 고용된 사람
③ 일용직 근로자 : 고용 계약기간이 1개월 미만인 사람 또는 일정한 사업장 없이 이동하며 일한 댓가를 받는 사람을 말함.
④ 자활근로/공공근로 : 구청 등에서 저소득층에게 일자리를 제공하여 근로의욕을 향상시켜 자립을 도와주기 위해 시행되는 임시 근로
⑤ 고용주 : 유급 종업원을 고용하여 경영하는 사업자
⑥ 자영업자 : 자기 혼자나 무급 가족의 힘으로 경영하는 사업
⑦ 무급가족종사자 : 가족이 경영하는 사업장에서 월급을 받지 않고 함께 일하는 사람

문 4) 월평균근로/사업 소득액 산출방법]

① 정규직 : 총 급여는 2008.10-12월 기간의 평균기본급여, 상여금, 초과근무수당, 경상적으로 매월 지급된 수당, 성과급을 모두 포함. 총 급여는 각종 세금, 보험기여금, 사회보장 부담금을 제외하기 전 금액.
② 계약직(임시·일용근로자) : 1년 동안 전체 소득을 12개월로 나눈 것을 작성.
③ 고용주·자영업자 : 순 소득은 연간 총매출액에서 연간 총 비용(재료비, 인건비, 월임대료, 광고비, 소모품비용 등)을 제외한 액수

번호	q05) 현재 직종		q06) 북한에서의 직업	q07) 제3국에서의 직업	q08) 비경제활동 이유	q09) 장애여부
	(예) 미취업자의 경우 직업명→없음 직업코드→00<br>※ [직종 중분류]에서 선택		**직접 기록**<br>※'노동자'응답 이외에 구체적으로 직종 작성	**직접 기록**<br>※구체적으로 작성 여러 가지일 경우 대표적인 것만 기로	① 근로 무능력 ② 근로의사 없음<br>③ 학업 ④ 가정일/양육<br>⑤ 구직활동 포기 ⑥ 기타<br>※ 사유가 겹치는 경우에 주요 이유 작성	① 있음<br>② 없음
	직업명 [s1]	번호[s2]				

[직종 중분류]

① 농업 및 임업	⑥ 건설업	⑪ 금융 및 보험업	⑯ 보건 및 사회복지사업
② 어업	⑦ 도매 및 소매업	⑫ 부동산 및 임대업	⑰ 오락, 문화 및 운동관련 서비스업
③ 광업	⑧ 숙박 및 음식점업	⑬ 공공행정, 국방 및 사회보장 행정	⑱ 기타 공공, 수리 및 개인 서비스업
④ 제조업	⑨ 운수업	⑭ 사업 서비스업	⑲ 가사 서비스업
⑤ 전기, 가스 및 수도 사업	⑩ 통신업	⑮ 교육 서비스업	⑳ 국제 및 외국기관

3. 4대 보험 (a3)

※ 아래 조사항목은 2008년 1년을 기준으로 응답해 주십시오. 비경제활동인구는 [4. 건강] 으로 이동하십시오.

번호	q01) 국민 연금	
	[s1] 가입대상	[s2] 미가입 이유
	① 만 15세 이상<br>② 국민연금 직장가입자<br>③ 국민연금 지역가입자<br>④ 국민공무원 및 사학연금가입자<br>⑤ 가입되어 있지 않음<br>⑥ 연금 수급자	① 보험료를 납부할 경제적 여유가 없어서<br>② 국민연금에 대한 불신 때문에<br>③ 가입의 필요성을 못 느껴서<br>④ 모름<br>⑤ 기타

번호	q02) 산재/고용보험		q03) 퇴직금 가입
	[s1] 산재보험 가입	[s2] 고용보험 가입	
	⓪ 비해당<br>① 가입<br>② 미가입<br>③ 모름	⓪ 비해당<br>① 가입<br>② 미가입<br>③ 모름	⓪ 비해당<br>① 가입<br>② 미가입<br>③ 모름

4. 건 강 (a4)

※ 작성하는 번호 순서는 1쪽 내용과 일치하도록 하십시오.

번호	q01) 건강 상태	q02) 1년간 진료 이용 횟수	q03) 1년간 입원 일수
	① 매우 건강하다<br>② 건강한 편이다<br>③ 보통이다<br>④ 건강하지 않은 편이다<br>⑤ 건강이 매우 안 좋다	(예) 20회→20<br>없음→ 0	(예) 10일→ 10<br>없음→ 0

번호	q04) 병원에 입원한 이유	q05) 만성질환	q06) 주요 병명
	⓪ 비해당 ① 질병<br>② 사고 ③ 출산<br><br>※ 병원에 입원한 적이 없으면 '⓪ 비해당'으로 표시. 병원에 입원한 경험이 있다면 **2008년 1년 동안 가장 장기간 병원에 입원한 경우를 기준**으로 작성.	⓪ 비해당<br>① 3개월 미만 투병·투약하고 있다<br>② 3~6개월 투병·투약하고 있다<br>③ 6개월 이상 투병·투약하고 있다<br><br>※ 투병 때문에 지속적인 투약이 필요하나 경제적인 사정에 의해서 못하고 있는 경우도 포함. 기간은 최초 투병 및 투약시점부터 계산	(예) 위염 → 3<br>없음 → 0<br><br>※ 아래 [질병 종류]를 참조하여 번호 기입. 중복 질환을 앓고 있는 경우 **주된 질환 한 가지**만 작성.

[문 7) 주요 병명' 기록시 유의사항]

- 감기와 같이 계절성 질환의 경우는 '⓪ 없음'으로 기록.
- 급성 질환의 경우는 주요 병명에 포함되지 않으며 '⑱ 기타 질병'으로 기록. 희귀난치성 질환의 경우는 '⑲ 희귀난치성 질환'으로 기록.

[질병 종류]

⓪ 없음	① 암(위, 간, 폐, 기관지등)	② 관절염, 요통, 좌골통, 디스크	③ 위염, 위궤양, 십이장궤양 등
④ 만성간염, 간경변	⑤ 당뇨병	⑥ 갑상선 질환	⑦ 고혈압, 저혈압
⑧ 중풍, 뇌혈관질환	⑨ 심근경색증, 협심증	⑩ 폐결핵, 결핵	⑪ 만성기관지염(심한 가래, 기침)
⑫ 천식	⑬ 백내장, 녹내장	⑭ 만성중이염	⑮ 만성신부전증(만성신장질환)
⑯ 골절, 탈골 및 사고 후유증	⑰ 골다공증	⑱ 기타 질병	⑲ 희귀난치성 질환

5. 소득 및 재산 (a5) (*)

* 소득 및 재산이 0인 경우에는 error가 아니라 0원으로 응답한 것임.

※ 2008.1.1～12.31 동안 다음에 해당하는 가족 전체의 소득은 얼마입니까?

		금 액	
재산소득(q01)	재산소득의 총 소득액은? (이자, 배당금, 임대료, 자격증 대여 등)	연간	만원
사회보험(q02)	사회보험의 총 소득액은? (공적연금, 고용보험, 산재보험 등)	연간	만원
민간보험(q03)	개인이 보험사에게서 받은 총 개인 연금액은? (개인연금)	연간	만원
따로 사는 가족으로부터의 보조금 (현금 등) (q04)	같이 살지 않는 부모/자녀/형제/친척으로 부터 받은 현금과 현물(현금 환산액)의 총 액수는?	연간	만원
민간보조금 (현금 및 현물) (q05)	친구나 이웃, 복지관, 종교·사회단체(학교 장학금 포함), 강연수입 등으로 받은 현금과 물건(현금 환산액) 보조금의 총 액수는?<br>※ 부모나 자녀로부터 받은 현금과 물건은 제외	연간	만원
국민기초생활보장급여 (q06)	정부(동사무소)로부터 받은 국민기초생활보장 수급자 급여의 총 액수는?<br>※ 쌀값 포함(일부 동사무소에서는 쌀값을 제외하고 현금을 지원하고 있으므로 이를 포함)	연간	만원
직업훈련 장려금 (q07)	6개월 이상 12개월까지 교육을 이수한 사람에게 월 수에 따라 20만원씩 지급	연간	만원
자격취득 장려금 (q08)	취업관련 자격증을 취득한 사람에게 200만원 지급	연간	만원
취업 장려금 (q09)	동일업체에서 1년 이상 연속 근무(4대 보험 가입)에 따라 장려금 지급	연간	만원

※ 없음 0

※ 귀 가족이 가지고 계신 전체 재산에 대한 질문입니다
(2008.12.31 기준, 가족 명의의 가계도 포함됩니다.)

			세부항목	금 액
1	소유 부동산 (q10)	2008.12.31 기준으로 소유부동산의 형태와 현재 가격은?	① [s1] 주택 (아파트, 단독주택 등)	만원
			② [s2] 주택외 건물 (가족명의의 가게 창고·상가 오피스텔 등)	만원
			③ [s3] 토지(주택지, 논, 밭, 산 등), 양식장, 기타 부동산	만원
2	점유 부동산 (q11)	2008.12.31 기준으로 점유부동산의 형태와 현재 가격은?	① [s1] 주택 전세보증금	만원
			② [s2] 가게 전세 보증금 준 것	만원
			③ [s3] 기타(권리금, 사업설비, 공장 기계, 가게 물건, 비닐하우스시설, 양식장 등)	만원
3	현금 자산 (q12)	2008.12.31 기준으로 가지고 있는 현금 자산의 형태와 가격은?	① [s1] 예금	만원
			② [s2] 적금(정기적금, 연금형 적금, 종신보험, 청약부금 등)	만원
			③ [s3] 주식·채권	만원
			④ [s4] 계돈 총액	만원
			⑤ [s5] 기타(남에게 빌려준 돈, 아파트 중도금(계약금) 부은 것 등)	만원

※ 없음 0

6. 빚 · 최저생계비 (a6)

※ 귀하 가족의 빚, 이자에 대한 질문입니다.
(2008. 12. 31기준, 사업용도의 부채는 제외)

			세부항목	금 액	
1	빚 형태 (q01)	2008.12.31 기준으로 빚은 얼마입니까?<br>※ 밀린 월세 등은 기타 빚에 포함.<br>※ 카드 할부 구매는 ③ 카드 빚에 작성, 현금 할부 구매는 ⑥ 기타 빚에 작성.	① [s1] 대출 (회사, 은행 마이너스통장 포함)		만원
			② [s2] 일반 사채		만원
			③ [s3] 카드 빚		만원
			④ [s4] 전세/임대 보증금 받은 돈		만원
			⑤ [s5] 외상, 미리 탄 계돈<br>※ 미리 탄 계돈의 경우 앞으로 부어야 하는 금액만 작성		만원
			⑥ [s6] 기타 빚(_______)<br>※ 브로커 비용 포함		만원
2	이자 (q02)	2008. 1년 동안 빚에 대해 지출한 이자는 총 액수는 얼마였습니까?	① [s1] 주거 관련 빚의 이자		만원
			② [s2] 기타 이자(주거 이자 제외)		만원

※ 없음 0

※ 귀 가족의 최저생계비에 대한 질문입니다.

		금 액		
3	귀하의 가족이 1달 동안 '근근이' 살아가는데 필요한 생활비는 얼마라고 생각하십니까? (q03)<br>※ 현금으로 지출되는 비용 + 자가 소비액 + 현물지원액	월평균		만원

A 설문지에 응답해주셔서 감사합니다.

B (개인 면담용)

1. 주 거 (b1)

※ 한국 입국 일로 부터 2008년 12월 31일 까지, 이사는 몇 번 하셨습니까? (q00)		번

구분	2007년												2008년											
	1	2	3	4	5	6	7	8	9	10	11	12	1	2	3	4	5	6	7	8	9	10	11	12
q01) 거주 기간<br>시작 : [s1]<br>끝 : [s2]<br>순서대로 t1-t4																								
q02) 거주 지역<br>순서대로 t1-t4<br>지역구분 [s1]<br>순서대로 t1-t4																								
q03) 주택 형태<br>순서대로 t1-t4																								
q04) 이사 이유<br>순서대로 t1-t4																								

[응답방법]

q01) **거주 기간**은 년, 월을 기준으로 하여 ⟷ 로 표시하십시오.
시작기간은 s1, 끝기간은 s2로 표시하였으며 2번 이사하였을 경우에는 t1, t2, t3까지 존재하게 됩니다.

q02) **거주 지역**은 시(군), 구(면), 동(리) 까지, 거주기간의 ⟷ 밑에 기입하여 주십시오.

q03) **주거 형태**는 아래 번호에서 1개를 선택하여 기입하여 주십시오.

① 국민임대주택 ② 전세 ③ 본인 소유 ④ 월세

⑤ 동거(본인 명의의 주택이 아닌 곳에 거주하고 있는 경우) ⑥ 양자(양부모) ⑦ 기타(직접 기록)

q04) **이사 이유**를 아래 번호에서 주된 원인 1개를 선택하여 기입하여 주십시오.

① 본인 또는 가족의 직장 때문에 ② 본인 또는 가족의 교육 때문에

③ 주거비용(전세금, 월세금 등)의 부담 때문에 ④ 거주 지역을 옮기고 싶어서

⑤ 기타(내용을 적어 주십시오.)

2. 취 업 (b2)

※ 한국 입국 일로 부터 2008년 12월 31일 까지 일한 직장이 모두 몇 곳이었습니까? (q00)		곳

구분	2007년												2008년											
	1	2	3	4	5	6	7	8	9	10	11	12	1	2	3	4	5	6	7	8	9	10	11	12
q01) 취업 기간																								
q02) 고용 형태																								
q03) 근무지 역할																								
q04) 직 원 수																								
q05) 근무 만족도																								
q06) 취업 경로																								
q07) 이직 이유																								

[응답방법]

q01) **취업기간**은 년, 월을 기준으로 하여 ↔ 로 표시하십시오. 예를 들어 2007년 1월 ~ 2007년 12월까지 취업하였다가 사직한 후 2008년 1월 ~ 2008년 6월 까지 취업, 2008년 10월 ~ 12월 취업, 2008년 2월 ~ 5월까지 겸업 경우 다음과 같이 표시합니다.

-위에 주거와 마찬가지로 취업시작기간은 s1, 끝난기간은 s2. t1부터 t6까지 차례대로 취업이동을 반영하였음.

q02) **고용형태**의 의미는 다음과 같습니다.
① 정규직 근로자 : 고용 계약기간이 1년 이상인 사람 또는 특별한 고용계약이 없어 기간이 정해져 있지 않더라도 계속 정규직원으로 일하면서 상여금, 수당 및 퇴직금 등을 받는 사람
② 계약직 근로자 : 고용 계약 기간이 1개월 이상 1년 미만인 사람 또는 일정한 사업 완료의 필요성에 의해서 고용된 사람
③ 일용직 근로자 : 고용 계약기간이 1개월 미만인 사람 또는 일정한 사업장 없이 이동하며 일한 댓가를 받는 사람을 말함.
④ 고용주 : 유급 종업원을 고용하여 경영하는 사업자
⑤ 자영업자 : 자기 혼자나 무급 가족의 힘으로 경영하는 사업
⑥ 무급가족종사자 : 가족이 경영하는 사업장에서 월급을 받지 않고 함께 일하는 사람

q03) **근무지 역할**을 다음 번호에서 1개 선택하십시오.
① 서비스 종사자(식당 종업원 등) ② 판매 종사자(영업직)
③ 농업, 임업, 어업의 경험 있는 기술자 ④ 기능원 및 관련 기능 종사자
⑤ 장치, 기계조작 및 조립 종사자(제조업 취업) ⑥ 단순 노무 종사자(노동 등)
⑦ 고위 직원 혹은 관리자(사업주 포함) ⑧ 해당 업무관련 전문가
⑨ 기술자 및 준전문가 ⑩ 사무 종사자

q04) **직원 수**를 다음 번호에서 1개 선택하십시오.
① 1인~4인 ② 5인~9인 ③ 10인~29인 ④ 30인~99인
⑤ 100인~299인 ⑥ 300인~499인 ⑦ 500인 이상

q05) **근무 만족도**를 다음 번호에서 1개 선택하십시오.
① 아주 만족 ② 약간 만족 ③ 약간 불만족 ④ 매우 불만족

q06) **취업경로**란 구직하는데 도움을 받은 개인이나 기관을 의미하며 **주요 이유 1개**를 선택하십시오.
① 생활정보지(벼룩시장 등) ② 인터넷 검색 ③ 다른 북한이주민의 소개 ④ 신변보호경찰관
⑤ 정부기관(고용지원노동센터 등) ⑥ 교회 및 민간단체 ⑦ 남한사람 ⑧ 기타(적을 것)

q07) **이직 주요 이유**를 다음 번호에서 1개 선택하십시오.
① 더 많은 월급을 받기 위해서 ② 더 높은 지위를 보장받기 위해서
③ 새로운 일을 배우고 싶어서 ④ 동료 또는 상사와 관계가 좋지 않아서
⑤ 회사의 사정으로(감원, 폐쇄 등) ⑥ 건강이 안 좋아서 쉬려고
⑦ 출산·양육 등을 위해서 ⑧ 개인적인 일로 장기간 출국을 해야 해서
⑨ 기타 (-------------------------------)

q08) 귀하는 2008년 12월 31일 기준으로 다음의 근로 형태 중 어디에 해당합니까? ☐
※ 가족 경제활동상태 A설문지 2쪽 문 2) 주된 경제활동 참여 상태'와 일치해야 함.

① 임금근로자
② 자영업, 고용주 **→ 문 6)로 이동**
③ 무급 가족 종사자 ——┘
④ 근로 능력 미취업자 → **문 8)로 이동**
⑤ 근로 무능력 미취업자 → **5쪽 [3. 교육] 으로 이동**

q09) **(모든 취업 응답자)** 2008년 1년간 일을 한 기간은 몇 개월입니까? 그리고, 일한 달의 평균 근로일수는 몇일 입니까?

[s1] 연간 총 ☐☐ 개월 **[s2]** 월 평균 근로일수 ☐☐ 일

[산출방법]

불규칙적으로 일한 경우, 하루라도 일한 달은 1개월로 간주. 예를 들어, 2008년 1년 중 3월에 2일, 4월에 15일, 7월과 8월에 20일 씩 일한 경우 '4개월'로, 일한 달의 평균 근로 일 수는 57일÷4개월=14.25일이므로 반올림 적용하여 '14일'로 기입

q10) 2008년 10월-12월 3개월의 기간 동안에, 주당 평균 근로시간은 몇 시간입니까?

[s1] 규칙적으로 일한 경우 : 주당 평균 [ ][ ] 시간

[s2] 불규칙적으로 일한 경우 : 일한 날의 하루 평균 [ ][ ] 시간

→ **5쪽 [3. 교육] 으로 이동**

※ 다음은 문 8) ~ 문 10)까지는 미취업자용 (근로능력) 질문입니다

q11) **(q08)**의 ④번 응답자만) 귀하는 2008년 12월 31일 기준으로 지난 4주 동안 돈을 벌 목적으로 일자리(사업)를 찾아보셨습니까? [ ]

① 그렇다 → **문8－1)로 이동** ② 아니다 → **문9)로 이동**

(q11)의 ①번 응답자만) 마지막으로 직장(사업)을 그만둔 후 총 구직 기간은 어느 정도였습니까?

총 구직 기간 [s1] [ ] 년 [s2] [ ][ ] 개월

(q11)의 ①번 응답자만) 일자리를 구하는 과정에서 다음과 같은 문제로 어려움을 경험하셨습니까?

구직 상의 어려움		전혀 그렇지 않다	그렇지 않다	그저 그렇다	그렇다	매우 그렇다
가족 특성 (q12)	[s1] 가정일 때문에	①	②	③	④	⑤
	[s2] 자녀를 돌보는 문제 때문에	①	②	③	④	⑤
	[s3] 가족을 간병해야 하기 때문에	①	②	③	④	⑤
	[s4] 가족(배우자, 부모 등)의 반대로	①	②	③	④	⑤

구직 상의 어려움		전혀 그렇지 않다	그렇지 않다	그저 그렇다	그렇다	매우 그렇다
개인 특성 (q13)	[s5] 나이 때문에	①	②	③	④	⑤
	[s6] 남녀 차별 때문에	①	②	③	④	⑤
	[s7] 외모 때문에	①	②	③	④	⑤
	[s8] 학력이 낮기 때문에	①	②	③	④	⑤
	[s9] 탈북자라는 이유 때문에	①	②	③	④	⑤
	[s10] 기술이나 기능이 부족해서	①	②	③	④	⑤
	[s11] 경력이 부족해서	①	②	③	④	⑤
	[s12] 건강 문제로	①	②	③	④	⑤
	[s13] 일자리에 대한 기대 수준이 높아서	①	②	③	④	⑤
	[s14] 신용 불량자라서	①	②	③	④	⑤
노동 시장 특성	[s15] 일자리가 없거나 부족해서	①	②	③	④	⑤
	[s16] 일자리에 대한 정보가 부족해서	①	②	③	④	⑤
	[s17] 근로조건이나 근로환경이 나빠서	①	②	③	④	⑤
	[s18] 급여 수준이 너무 낮은 일자리여서	①	②	③	④	⑤
	[s19] 고용이 불안정해서(비정규직이라서)	①	②	③	④	⑤

q13) 2008년 1년간 다음의 지원 프로그램에 참여한 경험이 있습니까? 참여하셨다면, 참여 기간과 만족도는 어느 정도였습니까? **※ 해당 프로그램 참여 경험이 '② 없다'면 참여기간과 만족도는 표기하지 않고, 5쪽 [3. 교육] 으로 이동.**

프 로 그 램	경험 여부 [s_1]		참여기간 [s_2]	만족도(※ 참여 경험이 있는 경우만 기록) [s_3]				
	있다	없다	※ 2008년 참여 개월 수<br>※ 참여 경험자만 기록	매우 불만족	불만족	그저 그렇다	만족	매우 만족
① 구직 알선 [s1_]	①	②	개월	①	②	③	④	⑤
② 직업 훈련 [s2_]	①	②	개월	①	②	③	④	⑤
③ 창업 지원 [s3_]	①	②	개월	①	②	③	④	⑤
④ 자활 공동체 [s4_]	①	②	개월	①	②	③	④	⑤

q14) **(q13)에서 하나라도 참여한 경험이 '① 있다'는 응답자만)** 귀하가 참여하신 지원 프로그램에 대해 어떻게 생각하십니까?

구 분	전혀 그렇지 않다	그렇지 않은 편이다	그저 그렇다	대체로 그런 편이다	매우 그렇다
① [s1] 구직(또는 창업)에 도움이 되었다.	①	②	③	④	⑤
② [s2] 직업능력 개발에 도움이 되었다.	①	②	③	④	⑤
③ [s3] 프로그램 참여로 인해 심리적 안정에 도움이 되었다.	①	②	③	④	⑤
④ [s4] 자신의 자존감 향상에 도움이 되었다.	①	②	③	④	⑤
⑤ [s5] 생계 유지에 도움이 되었다.	①	②	③	④	⑤

3. 교 육 (b3)

※ 한국 입국 일로 부터 2008년 12월 31일 까지, 한국에서 받았던 교육을 모두 적어 주십시오.
- 아래 각 문항별로 t1, t2, t3 차례대로 기록됨.

종 류	참여여부 [q0_]		가) 내용 [s1]	나) 기 간 [s2 : 시작, s3 : 끝]	다)취업/이직도움 정도 [s4]			
	예	아니오			①	②	③	④
q01) 정규학교	예	아니오	학교 전공	___년___월~___년___월	①	②	③	④
			학교 전공	___년___월~___년___월	①	②	③	④
			학교 전공	___년___월~___년___월	①	②	③	④
q02) 검정고시학원	예	아니오	과정	___년___월~___년___월	①	②	③	④
			과정	___년___월~___년___월	①	②	③	④
			과정	___년___월~___년___월	①	②	③	④
q03) 대안학교 (여명, 하늘꿈학교 등)	예	아니오	학교	___년___월~___년___월	①	②	③	④
			학교	___년___월~___년___월	①	②	③	④
			학교	___년___월~___년___월	①	②	③	④
문 4) 직업기술학교/학원 (자동차운전학원, 미용학원 자동차정비학원, 요리학원 컴퓨터학원, 중장비학원 등)	예	아니오	학원	___년___월~___년___월	①	②	③	④
			학원	___년___월~___년___월	①	②	③	④
			학원	___년___월~___년___월	①	②	③	④
문 5) 취득한 자격증 (자동차운전면허증, 미용사 자동차정비사, 한식·양식요리사 워드프로세스, 용접기술, 보일러, 제과·제빵, 간호사 등)	예	아니오	자격증	___년___월~___년___월	①	②	③	④
			자격증	___년___월~___년___월	①	②	③	④
			자격증	___년___월~___년___월	①	②	③	④
문 6) 기타	예	아니오		___년___월~___년___월	①	②	③	④
				___년___월~___년___월	①	②	③	④
				___년___월~___년___월	①	②	③	④

[응답방법]

가) 내용
문 1) 정규학교는 중·고등학교, 전문대, 대학교, 대학원 등을 의미합니다.
※ 중·고등학교에서 전공이 없을 경우 기록하지 않습니다.
문 4) 직업 기술학교/학원은 취직과 직접적으로 관련된 직업 기술교육을 의미합니다.
문 5) 취득한 자격증이 2개 이상일 경우 모두 적습니다.
나) 학교 또는 학원에 다닌 총 기간을 개월 단위로 적습니다.
다) 취업/이직 도움 정도는 해당하는 곳에 ○표 하십시오.
① 전혀 도움이 되지 않았다 ② 별로 도움이 되지 않았다 ③ 약간 도움이 되었다 ④ 매우 도움이 되었다.

4. 종 교 (b4)

q01) 귀하의 종교 생활에 대한 질문입니다. <u>한국 입국 일로 부터 2008년 12월 까지</u> 종교 활동에 참가하고 있다면, 말씀해주십시오.

구분 [s1]	2007년												2008년											
s2:시작 s3: 끝 t1~t4 순서대로	1	2	3	4	5	6	7	8	9	10	11	12	1	2	3	4	5	6	7	8	9	10	11	12
기독교																								
천주교																								
불 교																								
종교없음																								
기타( )																								

[응답방법]

※ 한국 입국일로 부터 2008년까지의 종교 활동을 화살표로 표시합니다.
※ 종교 생활이란 <u>정기적</u>으로 종교 활동에 참여하는 것을 의미합니다.

q02) 지금 종교 생활을 하고 있다면, 그것을 유지하는 가장 큰 이유 <u>2 개</u>를 순서대로 적어주십시오.

1 순위	[s1]	2 순위	[s2]

① 마음의 안정을 얻기 위해서
② 도덕적 삶을 사는데 도움이 되어서
③ 남한 사람들을 더 많이 알 수 있어서
④ 남한 생활에 필요한 정보를 얻을 수 있어서
⑤ 입국 과정에 도움을 준 사람이 원해서
⑥ 경제적 지원을 받을 수 있어서
⑦ 가족이 원해서
⑧ 믿음을 얻기 위해서
⑨ 다른 북한이주민을 만나기 위해서
⑩ 기타 (적을 것 : ______________________)

q03) 만약 교회(또는 다른 종교 기관)를 다니다가 나가지 않게 되었다면, 가장 큰 이유 <u>2 개</u>를 순서대로 적어주십시오.

1 순위	[s1]	2 순위	[s2]

① 교회(또는 다른 종교 기관)에서 말하는 교리를 믿으려 해도 믿어지지 않아서
② 신앙을 갖거나 예배, 모임에 참석하라고 강요당하는 것이 싫어서
③ 예배 의식이나 교리가 북한체제나 주체사상과 유사하여 거부감이 들어서
④ 목회자나 남한 교인들의 말과 행동이 다른 것에 실망해서
⑤ 남한 생활 적응에 실제적인 도움을 받을 수 없어서
⑥ 종교생활이 마음에 감동이 없어서
⑦ 직장생활이 바빠서
⑧ 설교가 이해되지 않아서
⑨ 기타 (적을 것 : ______________________)

5. 건 강 (b5)

※ 아래의 모든 조사 항목은 **2008년 1년간**을 기준으로 합니다.

q01) 귀하가 가지고 있는 건강보험은 다음 중 어디에 해당되십니까? [ ]

① 의료급여 → **q02)로 이동**
② 의료보험(직장)
③ 의료보험(지역) **[6. 법률]로 이동**
④ 없음
⑤ 기타

q02) **(q01)의 ①번 응답자만)** 의료급여 서비스에 얼마나 만족하십니까? [ ]

①---------- ②---------- ③---------- ④---------- ⑤
매 우 만 족 / 만족 / 보통 / 불만족 / 매 우 불만족

q03) **q02)의 ④,⑤번 응답자만)** 의료급여 서비스가 만족스럽지 않다면 가장 큰 이유가 무엇입니까? [ ]

① 혜택 범위가 좁은 것 (본인 부담이 많은 것) ③ 차별대우를 받음
② 적용 기간의 제한 ④ 매달 돈을 내야 하니까
⑤ 기타(직접 기록 : ______________________)

6. 법 률 (b6)

※ **2008년 1년간** 법적인 문제(민/형사상의 문제로 법원이나 경찰서 조사 등)를 경험한 적이 몇 번 있었습니까? (q00) [ ] 번

q01) 법적 문제의 내용	q02) 법적 해결 결과	q03) 법적 해결의 만족도 ① ② ③ ④ ⑤
t1부터 t3까지 차례대로		

[응답방법]

문 1) **법적 문제의 내용**은 구체적인 문제를 적는다. '**가해**'와 '**피해**'를 반드시 구분합니다.
예 : 교통사고 가해, 사기 피해, 폭력 가해 등
문 2) **법적 해결 결과**는 민/형사상 처벌, 벌금, 합의 등의 내용을 기입합니다.
문 3) **법적 해결의 만족도**는 법적 처리 결과를 받아들이는 정도입니다. 아래의 번호에서 1개를 선택하십시오.
① 전혀 만족할 수 없다 ② 만족하기 힘들다 ③ 그저 그렇다 ④ 대체로 만족한다 ⑤ 전적으로 만족한다

7. 심 리 (b7)

q01) 다음 질문에 해당되는 정도에 ○ 표 하십시오.

0=잘 모르겠다 1=없음 2=있기는 하나 문제가 되지 않음 3=문제가 됨

질 문		0	1	2	3
A	[s1] 죽음이나 신체적 위협을 주는 사건들을 자신이 경험했거나 타인의 경우를 목격한 적이 있다.				
	[s2] 당시 극심한 공포, 기운이 없거나, 고통을 경험하였다.				

q02) q01)에서 1가지 이상 해당되고 2점 또는 3점에 표시한 경우 구체적으로 그 내용을 적어 주십시오.

q03) 다음에 제시된 질문들은 고통스러운 사건을 경험한 후에 사람들에게 나타날 수 있는 증상들입니다. 질문을 주의 깊게 읽고 <u>지난 1 주일</u> 동안 얼마나 괴로웠는지 표시해 주십시오.

질 문		0	1	2	3
B	[b1] 고통스러웠던 과거의 사건이 집요하게 떠오르는 것을 경험하였다.				
	[b2] 고통스러웠던 과거의 사건에 대해 반복적이고 괴로운 꿈에 시달렸다.				
	[b3] 마치 과거의 사건이 다시 일어나는 듯 한 느낌을 경험하였다.				
	[b4] 고통스러웠던 과거의 사건이 무언가에 의해 기억날 때 극심한 심리적 고통을 느꼈다.				
	[b5] 고통스러웠던 과거의 사건이 무언가에 의해 기억날 때 진땀이 나거나 극심한 공포와 무력감을 느꼈다.				
C	[c1] 고통스러웠던 과거의 사건과 관련된 생각이나 느낌, 대화 등을 일부러 피하였다.				
	[c2] 고통스러웠던 과거의 사건을 기억나게 하는 행동이나 장소, 사람 등을 일부러 피하였다.				
	[c3] 과거 사건의 가장 고통스러웠던 부분을 기억할 수가 없었다.				
	[c4] 중요한 활동에 대한 흥미나 참여가 매우 줄어들었다.				
	[c5] 타인으로부터 외면 받거나 멀어지는 듯 한 느낌이다.				
	[c6] 어떤 일에 대해 감정을 느끼는 것이 무뎌지고 둔해졌다.				
	[c7] 나에게는 미래가 없는 듯 한 느낌이 들었다.				
D	[d1] 잠들기 어렵거나 잠을 계속 자는 것이 어려워졌다.				
	[d2] 평소와 달리 예민해지거나 갑자기 화를 버럭 낸 적이 있다.				
	[d3] 어떤 일에 집중하기가 어려워졌다.				
	[d4] 특별한 이유 없이 지나치게 경계하거나 조심스러워졌다.				
	[d5] 갑작스런 소리에 움찔하거나 쉽게 놀라게 되었다.				

q04e) 기 간

질 문		0	1	2	3
E	B,C,D에 있는 증상들이 1개월 이상 지속되었다.				

q05f) 생활 장해

질 문		0	1	2	3
F	B, C, D에서 경험한 증상 때문에 정신적·신체적으로 심각한 고통을 경험하였거나 사회 활동, 직장 생활 및 다른 중요한 생활에서 지장을 받았다.				

8. 대인관계 (b8)

※ 사람들은 살아가면서 어려운 일이 생겼을 때 아는 사람의 **도움**을 필요로 합니다. **하나원 수료 이후** 여러 가지 이유로 아는 분들의 도움을 받으신 경우를 생각해 주십시오. **사회복지기관, 공공기관, 교회 등에서 받은 도움뿐만 아니라 가족, 그리고 친구나 아는 사람들에게 도움을 받은 경우를 모두** 떠올려 주세요.

※ 그러나 스스로의 노력으로 정보를 얻은 것(신문이나 인터넷을 통해 얻은 정보)은 제외됩니다.

q01) 아래와 같은 도움을 받으셨습니까?

내 용	도움을 받지 못했다	도움을 받았다
구직 정보 도움 [s1]	①	②
교육 정보 도움 [s2]	①	②
생활 정보 도움 [s3]	①	②
일상 생활 도움 [s4]	①	②
정신적인 도움 [s5]	①	②
금전적인 도움 [s6]	①	②
물질적인 도움 [s7]	①	②

※ 문 1)을 모두 질문한 후 문 2)로 이동.

q02) q01)의 도움이 필요하셨습니까?

필요하지 않았다	필요했다
①	②
①	②
①	②
①	②
①	②
①	②
①	②

- 이상의 문항은 q01s_은 도움을 받은 정도, q02s_은 필요한 정도로 구성되어 있음.

q03) 아래와 같은 도움을 얼마나 받으셨습니까?

구 분	필요한 것 보다 **훨씬 적게** 받았다	필요한 것 보다 **적게** 받았다.	필요한 만큼 받았다	필요한 것 보다 **많이** 받았다	필요한 것 보다 **훨씬 많이** 받았다
구직 정보 도움 [s1]	①	②	③	④	⑤
교육 정보 도움 [s2]	①	②	③	④	⑤
생활 정보 도움 [s3]	①	②	③	④	⑤
일상 생활 도움 [s4]	①	②	③	④	⑤
정신적인 도움 [s5]	①	②	③	④	⑤
금전적인 도움 [s6]	①	②	③	④	⑤
물질적인 도움 [s7]	①	②	③	④	⑤

[응답 설명]

1. **구직 정보 도움** : 아는 분이 '어디서 사람을 구하더라', '어디서 보니 여기서 일자리를 구할 수 있더라', '거기서 일하려면 이런 걸 준비해야 하고 이런 사람을 만나야 한다' 등과 같은 정보입니다.

2. **교육 정보 도움** : 아는 분이 '어느 학교를 가야 하는지', '어느 학원을 가야 하는지', '검정고시를 보려면 어떻게 해야 하는지'와 같은 정보에 대한 도움을 의미합니다.

3. **생활 정보 도움** : 아는 분이 '어디서 핸드폰을 싸게 살 수 있는지', '어느 병원을 가야 하는지', '물건들을 어디서 구입해야 하는지' 등의 정보입니다.

4. **일상 생활 도움 :** 아는 분이 밥이나 반찬을 시간 내어 직접 해주신 경우, 아이가 있을 때는 아이를 대신 돌봐준 일, 자동차를 태워 준 일 등을 의미합니다.

5. **정신적인 도움 :** 여러 가지 문제에 부딪히셨을 때, 금전적, 물질적 도움은 아니지만 옆에서 마음을 위로해주거나 고민거리를 들어 주는 일을 의미합니다.

6. **금전적인 도움 :** 귀하에게 돈을 빌려주거나 돈을 대 주는 것을 의미하며 용돈도 포함됩니다.

7. **물질적인 도움 :** 친구나 가족 혹은 아는 사람이 귀하에게 가구나 옷 등과 같은 생활에 필요한 물건들을 제공해 주는 것을 의미합니다.

q04) 하나원을 수료하신 이후 위에서 말씀하신 도움을 주신 분들은 총 몇 분이십니까?
※ 위에서 말씀하신 분들을 가능한 많이 떠올려주십시오. 조금이라도 도움을 주신 분들은 모두 포함시켜 주십시오.

	명

▼ 아래의 네트워크 문항은 각각이 변수화 되어 있음. 예를 들어, 도움을 준 사람이 3명일 경우에는 변수명 뒤에 h1은 첫 번째 사람, h2는 두 번째 사람, h3는 세 번째 사람에 대한 각각의 정보를 나타냄. 따라서 2번째 사람의 북한학력은 b8q05s41h2 로 확인가능함.

가장 많은 도움을 준 순서대로 말씀해주십시오. 먼저 그분들의 별명이나 성(이름)을 우선 말씀해주시고 자세한 사항을 질문하겠습니다.

q05) ※ 도움을 주신 분이 7명 이상일 경우 도움을 많이 주신 분들부터 말씀해 주십시오.

번호	[s1] 성별	[s2] 나이	[s3] 출신	학력		현재 직업 [s5]	소득 [s6]	거주지 [s7]
				북한 [s41]	남한 / 기타 [s42]			
순서대로 기입	①남<br>②여	( )세	① 남한<br>② 북한<br>③ 조선족<br>④ 한족<br>⑤ 기타	⓪ 모름<br>① 미취학 (만 7세 이하)<br>② 무학 (만 8세 이상)<br>③ 인민중퇴<br>④ 인민졸업<br>⑤ 고등중 중퇴<br>⑥ 고등중 졸업<br>⑦ 대학교 중퇴<br>⑧ 대학교 졸업<br>⑨ 대학원 이상	⓪ 모름<br>① 없음<br>② 미취학<br>③ 초등 재학/졸업<br>④ 중등 재학/졸업<br>⑤ 고등 재학/중퇴<br>⑥ 고등 졸업<br>⑦ 대학 재학/중퇴<br>⑧ 대학 졸업<br>⑨ 고입검정합격<br>⑩ 대입검정합격<br>⑪ 대학원 재학/졸업	① 있음<br>② 없음	①남한 일반인에 비해 매우 못 산다<br>②남한 일반인에 비해 못 산다<br>③남한 일반인과 비슷하다<br>④남한 일반인에 비해 잘 산다<br>⑤남한 일반인에 비해 매우 잘 산다	( )시/군<br>[s70] 16개 광역시/도로 나눈 것

q06) 도움을 주신 분들과 귀하의 관계에 대해 질문 드리겠습니다.
(도움 준 사람이 가족인 경우에도 모두 답하여 주십시오)

번호	[s1] 관계	[s2] 만나게 된 계기	[s3] 만나게 된 시점	도움준 일 각문항당 도움을 줬을 경우 1 아니면 0	[s5] 만나는 횟수	[s6] 연락하는 횟수	[s7] 친한 정도
순서대로 기입	*응답설명 참조<br>기타의 경우에는 [rela2_help]	①가족<br>②종교기관<br>③신변보호관<br>④사회복지관<br>⑤적십자<br>⑥동네<br>⑦학교<br>⑧아는 사람의 소개로<br>⑨탈북 과정에서<br>⑩하나원<br>⑪자녀들의 학교<br>⑫브로커<br>⑬기타( )<br>-> [firstmeet2_help]<br>⑭직장상사	①북한<br>②제3국 탈북 과정<br>③하나원 이전<br>④하나원 이후	①구직 정보 [s41]<br>②교육 정보 [s42]<br>③생활 정보 [s43]<br>④실질적인 도움 [s44]<br>⑤감정적인 도움 [s46]<br>⑥금전적인 도움 [s47]<br>⑦물질적인 도움 [s48]<br>※ 해당 번호 모두 기입	①거의 매일<br>②일주일에 한, 두 번<br>③한 달에 한번 정도<br>④몇 달에 한 번 정도<br>⑤1년에 1-2번 정도	①거의 매일<br>②일주일에 한, 두 번<br>③한 달에 한번 정도<br>④몇 달에 한 번 정도<br>⑤1년에 1-2번 정도<br>※전화, 문자, 이메일, 채팅 등을 포함.	①전혀 친하지 않다<br>②친하지 않다<br>③보통이다<br>④친하다<br>⑤매우 친하다

[응답 설명]
※ 가족 및 친척 : ① 남편 ② 아내 ③ 아버지 ④어머니 ⑤ 아들 ⑥ 딸 ⑦ 형제 ⑧ 자매 ⑨ 친척
※ 기관 관계자 : ⑩ 사회복지사 ⑪ 신변보호관 ⑫ 거주지보호관 ⑬ 취업보호관 ⑭ 하나원공무원 ⑮ 정착도우미 ⑯ 학교교사 ⑰ 종교관계자
※ 친구 및 이웃 : ⑱ 북한이주민친구 ⑲ 직장 친구 ⑳ 학교 친구 ㉑ 종교기관 친구 ㉒ 고향 친구 ㉓ 아파트 이웃 ㉔ 기타 (직접 기록)

h_h_) 도움을 주신 분들이 서로 얼마나 알고 있는지 질문하겠습니다. 이름 1과 이름 2가 어느 정도 아는 사이인지를 '① 전혀 모른다 ② 잘 모른다 ③ 보통이다 ④ 잘 안다 ⑤ 매우 잘 안다' 중에서 선택하십시오.

	이름 1:	이름 2:	이름 3:	이름 4:	이름 5:	이름 6:	이름 7:
이름 1:							
이름 2:							
이름 3:							
이름 4:							
이름 5:							
이름 6:							
이름 7:							

▼ 마지막 관계 문항의 경우에는 h_h_ 로 표시됨. 예를 들어 1번사람과 2번 사람이 얼마나 아는지의 관계를 알기 위해서는 h1_h2의 변수로 확인 가능. 3번과 5번 사람의 경우에는 h3h5 로 확인가능.

B 설문지에 응답해주셔서 감사합니다.

이 조사표에 기재된 내용은 통계법 제13조에 의하여 비밀이 보장됩니다

북한이주민 남한사회 적응 변화조사

C
(개인 설문용)

2009년

2009 북한이주민 연구팀

조 사 위 원	(인)	조사 일시	2009.　　.　　.	응답자 성명	
설문완료 확인	1. 완료　2. 미완	보완 사항	1. 완료　2. 미완	담당 연구원	(인)

1. 생활 경험 (c1)

※ [q01] 다음은 귀하께서 대한민국 생활에서 겪을 수 있는 일들입니다. 만약 지난 2008년 1년 동안 다음과 같은 일을 경험하셨다면 '예'에 없었다면 '아니오'에 ○표 하십시오. [예 -> 1, 아니오 ->2]

번호	문 항	예	아니오
s1	취업을 했다.	예	아니오
s2	북한이나 제 3국에 있는 가족의 소식을 들었다.	예	아니오
s3	직장을 그만두었다.	예	아니오
s4	이사를 했다.	예	아니오
s5	사기를 당하였다.	예	아니오
s6	결혼이나 동거를 했다.	예	아니오
s7	이혼이나 별거를 했다.	예	아니오
s8	심한 질병이나 신체적인 상처를 입었다.	예	아니오
s9	가족이나 가까운 친지가 남한에 입국했다.	예	아니오
s10	법을 위반하여 처벌을 받았다.	예	아니오
s11	가족이 실종되거나 북한으로 송환되었다.	예	아니오
s12	가까운 가족이나 친척이 사망하였다.	예	아니오

※ [q02] 다음은 귀하께서 남한 생활에서 겪을 수 있는 스트레스입니다. 만약 지난 2008년 1년 동안 다음과 같은 스트레스를 경험했다면 그 정도에 ○표 하십시오. 경험한 적이 없으시면 해당 없음 '⓪'에 ○표 하십시오.

번호	문 항	전혀 없음	약간 있음	상당히 있음	매우 있음	해당 없음
s1	남한 언어를 잘 이해하지 못한다.	①	②	③	④	⓪
s2	음식, 옷차림, 주택 등 생활 조건들이 달라 어렵다.	①	②	③	④	⓪
s3	남한 사람들의 사고방식을 이해하기가 힘들다.	①	②	③	④	⓪
s4	북한에서의 가치관이나 생활습관이 여기서 잘 맞지 않는다는 느낌이 든다.	①	②	③	④	⓪
s5	나의 경제 사정이 나쁘다.	①	②	③	④	⓪
s6	북한이주민에 대한 차별과 편견을 경험하였다.	①	②	③	④	⓪
s7	남한 사회에서 살아가는데 필요한 정보가 부족하다.	①	②	③	④	⓪
s8	북한에 두고 온 가족들 생각에 마음이 아프다.	①	②	③	④	⓪
s9	내가 무능하다는 느낌이 든다.	①	②	③	④	⓪
s10	내가 남한 사회 속에서 살고 있다는 느낌이 들지 않는다.	①	②	③	④	⓪
s11	내가 북한출신임이 주변에 알려져서 불편하다.	①	②	③	④	⓪

※ [q03] 귀하는 일상생활에서 얼마나 자주 다음과 같은 차별을 남한사람들로 부터 경험하십니까? 자신의 상황을 가장 잘 나타내는 곳에 ○표 하십시오.

번호	내 용	전혀 경험하지 않음	좀처럼 경험하지 않음	때때로 경험함	자주 경험함
s1	나를 열등한(남보다 못한) 사람처럼 대한다.	①	②	③	④
s2	나를 똑똑하지 않은 사람처럼 대한다.	①	②	③	④
s3	나를 두려워하는 것처럼 행동한다.	①	②	③	④
s4	남들에게 대하는 것처럼 나에게 예의를 갖추지 않는다.	①	②	③	④
s5	남들에게 대하는 것처럼 나를 존중하지 않는다.	①	②	③	④
s6	음식점이나 가게에서 소홀하게 대접받았다.	①	②	③	④
s7	나를 정직하지 못한 사람처럼 대한다.	①	②	③	④
s8	나에게 욕하거나 모욕을 한다.	①	②	③	④
s9	나를 위협하거나 괴롭힌다.	①	②	③	④

※ [q04] 귀하는 지난 1년간 다음과 같은 일을 경험하신 적이 있습니까? 다음과 같은 일을 경험하셨다면 '예'에 ○표 하십시오. 다음과 같은 일을 경험했지만 차별에 의한 것이 아니라면 '아니오'에 ○표 하십시오. [예->1, 아니오->2]

번호	내 용	예	아니오
s1	차별에 의해 취업이 되지 않았다.	예	아니오
s2	차별에 의해 승진되지 않았다.	예	아니오
s3	차별에 의해 직장에서 해고당하였다.	예	아니오
s4	차별에 의해 이사 가라는 압력을 받았다.	예	아니오

2. 의 식 (b2)

※ [q01] 다음은 대한민국 사회에 대한 귀하의 생각을 묻는 질문입니다. 자신의 의견과 가장 일치하는 곳 에 ○표 하십시오.

번호	내 용	전혀 아니다	아니다	보통이다	그렇다	매우 그렇다
s1	지나치게 복잡하다.	①	②	③	④	⑤
s2	사회가 혼란스럽다.	①	②	③	④	⑤
s3	안전하지 못하다.	①	②	③	④	⑤
s4	부정부패가 심하다.	①	②	③	④	⑤
s5	믿을 수 없다.	①	②	③	④	⑤
s6	돈이면 안 되는 일이 없다.	①	②	③	④	⑤
s7	너무 권위적이다.	①	②	③	④	⑤
s8	남을 배척한다.	①	②	③	④	⑤
s9	습관화된 생활 방식에 얽매여 있다.	①	②	③	④	⑤
s10	인맥이 있어야 성공한다.	①	②	③	④	⑤
s11	지역 감정이 심하다.	①	②	③	④	⑤
s12	학벌이 좋아야 한다.	①	②	③	④	⑤
s13	빈부격차가 심하다.	①	②	③	④	⑤
s14	사람에 대한 차별이 있다.	①	②	③	④	⑤
s15	남녀차별이 있다.	①	②	③	④	⑤
s16	대한민국은 발전가능성이 있다.	①	②	③	④	⑤
s17	개선을 위해 노력중이다.	①	②	③	④	⑤
s18	살기 좋아질 것이다.	①	②	③	④	⑤
s19	선진국이다.	①	②	③	④	⑤
s20	세계를 주도하는 강대국이다.	①	②	③	④	⑤
s21	세계질서와 정의를 수호하는 나라이다.	①	②	③	④	⑤
s22	자유를 누릴 수 있다.	①	②	③	④	⑤
s23	다양한 생각이 받아들여진다.	①	②	③	④	⑤
s24	생활이 자유분방하다.	①	②	③	④	⑤
s25	민주적인 사회이다.	①	②	③	④	⑤
s26	기회가 공평하게 주어진다.	①	②	③	④	⑤
s27	모든 사람이 평등하다.	①	②	③	④	⑤
s28	합리적인 사회이다.	①	②	③	④	⑤
s29	공적인 일과 개인적인 일을 잘 구분한다.	①	②	③	④	⑤
s30	법과 질서가 분명하게 지켜진다.	①	②	③	④	⑤

※ [q02] 다음은 귀하가 대한민국 국민이라는 것에 대한 생각과 느낌을 묻는 질문입니다. 자신의 상황과 가장 일치하는 곳에 ○표 하십시오.

번호	내 용	전혀 아니다	아니다	그렇다	매우 그렇다
s1	나는 대한민국 사람이라는 것이 기분이 좋다.	①	②	③	④
s2	나는 대한민국의 전통을 자랑스럽게 여긴다.	①	②	③	④
s3	나는 대한민국에 대해서 별로 자랑할 것이 없다고 생각한다.	①	②	③	④
s4	나는 대한민국 사람이라는 것에 만족한다.	①	②	③	④
s5	나는 대한민국 사람들과 일체감을 느낀다.	①	②	③	④
s6	나는 대한민국 국민과 더불어 함께 살아간다는 느낌이 있다.	①	②	③	④
s7	나는 대한민국에 대한 주인의식을 갖고 있다.	①	②	③	④
s8	나는 대한민국에서 내가 쓸모없는 사람이라는 생각이 든다.	①	②	③	④
s9	나는 내가 대한민국 국민이라는 사실이 싫다.	①	②	③	④
s10	나는 마음으로 대한민국 사람이라는 생각이 든다.	①	②	③	④
s11	나는 대한민국에서 외국인 같다는 생각이 든다.	①	②	③	④
s12	나는 앞으로 대한민국에서 계속 살 생각이다.	①	②	③	④
s13	나는 대한민국에 익숙해져야 한다는 것이 거북하다.	①	②	③	④
s14	나는 대한민국 국민으로서의 모습을 갖추어가고 있다.	①	②	③	④
s15	내가 대한민국 국민이라는 것은 내 인생에서 중요하다.	①	②	③	④
s16	나는 지금 대한민국에 사는 것이 좋다.	①	②	③	④
s17	나는 대한민국이 북한과 축구를 할 때 대한민국을 응원한다.	①	②	③	④
s18	대한민국이 잘 되어야 나도 잘 된다고 생각한다.	①	②	③	④
s19	나는 노력하여 대한민국을 발전시키고 싶다.	①	②	③	④
s20	대한민국이 잘되면 내가 잘되는 것처럼 기쁘다.	①	②	③	④

▶▶▶ 5쪽에서 계속

번호	내 용	전혀 아니다	아니다	그렇다	매우 그렇다
s21	누군가 대한민국에 대해서 비난을 하면 내가 비판을 받는 것 같다.	①	②	③	④
s22	나는 외국 사람들이 대한민국에 대해 어떻게 생각하는지에 관심이 많다.	①	②	③	④
s23	내가 대한민국 국민이라는 사실은 내 사고와 행동에 큰 영향을 준다.	①	②	③	④
s24	나는 통일이 되면 북한에 가서 살고 싶다.	①	②	③	④
s25	내가 대한민국 문화에 속해 있다고 느낀다.	①	②	③	④
s26	나는 내가 '대한민국' 사람이라는 의식이 강하다.	①	②	③	④
s27	나는 대한민국 사람이라는 것이 자랑스럽다.	①	②	③	④
s28	나는 꼭 대한민국 사람과 결혼하고 싶다.	①	②	③	④
s29	나는 앞으로 선거(대통령, 국회위원, 지방자치단체장 선거)가 있을 때 투표에 참여할 것이다.	①	②	③	④

※ [q03] 다음은 귀하의 대한민국에 대한 지식 정도를 묻는 질문입니다. 자신의 상황과 가장 일치하는 곳에 ○표 하십시오.

번호	내 용	전혀 아니다	아니다	그렇다	매우 그렇다
s1	대한민국에 어떤 위인들이 있는지 알고 있다.	①	②	③	④
s2	대한민국에서 어떤 TV프로그램이 인기 있는지 알고 있다.	①	②	③	④
s3	대한민국의 주요 신문과 잡지가 어떤 것인지 알고 있다.	①	②	③	④
s4	대한민국의 유명한 배우들로는 누가 있는지 알고 있다.	①	②	③	④
s5	대한민국의 역사를 알고 있다.	①	②	③	④
s6	대한민국의 정치 지도자들 중에 어떤 사람이 있는지 알고 있다.	①	②	③	④

※ [q04] 다음은 귀하의 남북한 사회에 대한 태도를 묻는 질문입니다. 자신의 태도를 가장 잘 나타내는 곳에 ○표 하십시오.

번호	내 용	전혀 그렇지 않다	조금 그렇다	대체로 그렇다	전적으로 그렇다
s1	남한에서 살고 있지만 정신적 뿌리는 북한이라는 생각을 잊지 않으려고 노력한다.	①	②	③	④
s2	나는 북한에서 배운 지식과 기술이 가치 있다고 생각한다.	①	②	③	④
s3	나는 진정한 남한 사람이 되려고 노력하고 있다.	①	②	③	④
s4	나는 자식들이(여기서 같이 산다면) 북한의 문화를 잊지 않도록 가르치겠다.	①	②	③	④
s5	나는 북한 이주민들과 계속 긴밀한 관계를 유지하려고 노력한다.	①	②	③	④
s6	나는 남한에서 경험한 새로운 생활이 매우 유익하고 재미있다고 생각하면서 지내려고 한다.	①	②	③	④
s7	북한에 남아 있는 아름다운 전통문화를 계속 발전시켜야 한다고 생각한다.	①	②	③	④
s8	나는 북한에서의 삶을 자주 떠올리며 그리워한다.	①	②	③	④
s9	나는 북한이주민끼리 만난 자리에서는 북한식 말투를 써야 한다고 생각한다.	①	②	③	④
s10	나는 남한사회의 생활양식을 받아들인다.	①	②	③	④
s11	나는 일상생활에서 남한식 말투를 사용하려고 노력한다.	①	②	③	④
s12	북한출신 사람들의 모임에 나가면 마음이 편안해지는 느낌이 든다.	①	②	③	④
s13	나는 남한에서 잘살기 위해서라면 무엇이든지 해보겠다.	①	②	③	④
s14	나는 자녀들이 여기에서(같이 살거나 산다면) 남한 사회의 문화를 잘 받아들이도록 해주겠다.	①	②	③	④
s15	나는 남한 사회에 대해 즐거운 마음으로 배우려고 한다.	①	②	③	④
s16	나는 새롭게 사귄 남한 사람들과 허물없이 친하게 지내려고 노력한다.	①	②	③	④

※ [q05] 다음은 대한민국 정치에 대한 귀하의 생각과 느낌에 관한 것입니다. 자신의 의견과 가장 일치하는 곳에 ○표 하십시오.

번호	내 용	전혀 안 그렇다	별로 안 그렇다	보통이다	어느 정도 그렇다	매우 그렇다
s1	나는 평소에 정치에 대해서 주위 사람들과 대화를 나누고 있다.	①	②	③	④	⑤
s2	나는 TV 또는 신문의 정치 기사를 관심 있게 보고 읽는다.	①	②	③	④	⑤
s3	각종 공직선거에서 내가 던진 한 표가 한국의 정치발전에 중요한 영향을 미친다고 생각한다.	①	②	③	④	⑤
s4	내게 기회가 주어진다면 각종 공직선거에 나가는 등 적극적으로 정치에 참여할 의향이 있다.	①	②	③	④	⑤
s5	나와 같은 보통 사람은 정부가 하는 일에 대해 말할 지식이나 능력이 없다.	①	②	③	④	⑤
s6	정치나 정부가 하는 일은 너무 복잡해서 어떻게 돌아가는지 알 수가 없다.	①	②	③	④	⑤
s7	사회의 변화와 발전을 위해서는 촛불시위 등과 같은 적극적인 사회 참여가 필요하다.	①	②	③	④	⑤
s8	정부 관리는 일반 국민이 무엇을 원하는지 거의 관심이 없는 것 같다.	①	②	③	④	⑤
s9	나는 복잡하긴 하지만 다양성과 차이를 고려하는 대한민국식 민주주의를 선호한다.	①	②	③	④	⑤
s10	북한이주민이 국회의원이나 시의원에 출마한다면 나는 지지하겠다.	①	②	③	④	⑤

※ [q06] 귀하가 평소 정치적 지식이나 정보를 얻는데 있어서, 다음 방법에서 가장 도움을 받고 있다고 생각하는 것 2개를 골라 번호를 써주십시오.

[s1]	[s2]

① 신문 ② 인터넷 ③ TV ④ 라디오
⑤ 북한이주민 단체 ⑥ 주위 북한이주민 ⑦ 주위 남한사람 ⑧ 기타 ____________

3. 만 족 도 (c3)

※ [q01] 다음은 북한이주민 정책 만족도에 대한 질문입니다. 귀하의 의견과 가장 일치하는 곳에 ○표 하십시오.

번호	내 용	전혀 안 그렇다	별로 안 그렇다	보통 이다	어느 정도 그렇다	매우 그렇다
s1	정부의 북한이주민 지원 정책 중 주택지원 내용에 만족한다	①	②	③	④	⑤
s2	정부의 북한이주민 지원 정책 중 교육지원 내용에 만족한다	①	②	③	④	⑤
s3	정부의 북한이주민 지원 정책 중 의료지원 내용에 만족한다	①	②	③	④	⑤
s4	정부의 북한이주민 지원 정책 중 취업지원 내용에 만족한다	①	②	③	④	⑤
s5	정부의 북한이주민 지원 정책 중 정착지원금 내용에 만족한다	①	②	③	④	⑤
s6	정부의 북한이주민 지원 정책에 대하여 전체적으로 만족한다	①	②	③	④	⑤

※[q02] 다음은 북한이주민에 대한 생각과 생활 만족도에 대한 질문입니다. 귀하의 의견과 가장 일치하는 곳에 ○표 하십시오.

번호	내 용	전혀 안 그렇다	별로 안 그렇다	보통 이다	어느 정도 그렇다	매우 그렇다
s1	일반적으로 북한이주민들은 스스로 자립하려는 의지가 부족하다.	①	②	③	④	⑤
s2	북한이주민들끼리 힘과 의견을 모을 수 있는 더 큰 단체가 필요하다고 생각한다.	①	②	③	④	⑤
s3	북한이주민들끼리의 단체가 생긴다면 나는 적극적으로 참여하겠다.	①	②	③	④	⑤
s4	현재 남한에서의 나의 생활 전체에 대하여 만족한다.	①	②	③	④	⑤

※[q03] 다음은 귀하의 삶에 대한 만족도 질문입니다. 자신의 의견과 가장 일치하는 곳에 ○표 하십시오.

번호	내 용	전혀 아니다	대체로 아니다	조금 아니다	중간 이다	조금 그렇다	대체로 그렇다	아주 그렇다
s1	대체로 현재 나의 생활은 내가 바라던 생활과 비슷하다.	①	②	③	④	⑤	⑥	⑦
s2	내 생활 환경은 훌륭하다.	①	②	③	④	⑤	⑥	⑦
s3	나는 나의 생활에 만족한다.	①	②	③	④	⑤	⑥	⑦
s4	지금까지 나는 내가 원하던 중요한 일을 이루었다.	①	②	③	④	⑤	⑥	⑦
s5	나는 다시 태어난다고 해도 지금까지의 생활방식을 바꾸지 않을 것이다.	①	②	③	④	⑤	⑥	⑦

4. 건 강 (c4)

※ [q01] 다음 문항들은 일상생활에서 흔히 느낄 수 있는 경험들을 열거한 것입니다. 각 항목을 주의 깊게 읽고 <u>**오늘을 포함해서 지난 1주일 동안**</u> 귀하의 상태를 가장 잘 나타낸 곳에 ○표 하십시오.

번호	내 용	전 혀 그렇지 않았다	조 금 그렇다	상당히 그렇다	심하게 그렇다
s1	기운이 없고 침체된 기분이다.	⓪	①	②	③
s2	죽고 싶은 생각이 든다.	⓪	①	②	③
s3	울기를 잘한다.	⓪	①	②	③
s4	어떤 함정에 빠져 헤어 나올 수 없는 기분이다.	⓪	①	②	③
s5	자책을 잘 한다.	⓪	①	②	③
s6	외롭다.	⓪	①	②	③
s7	기분이 울적하다.	⓪	①	②	③
s8	매사에 걱정이 많다.	⓪	①	②	③
s9	매사에 관심과 흥미가 없다.	⓪	①	②	③
s10	장래에 희망이 없는 것 같다.	⓪	①	②	③
s11	매사가 힘들다.	⓪	①	②	③
s12	자신이 무가치하다는 생각이 든다.	⓪	①	②	③
s13	입맛이 없다.	⓪	①	②	③
s14	잠이 들거나 깊이 자는 것이 어렵다.	⓪	①	②	③
s15	성욕과 쾌감이 감소하였다.	⓪	①	②	③
s16	이유없이 갑자기 무서울 때가 있다.	⓪	①	②	③
s17	두려움을 느낄 때가 있다.	⓪	①	②	③
s18	기절하거나, 어지럽거나, 기운이 없어질 때가 있다.	⓪	①	②	③
s19	초조하고 속에서 떨리는 느낌이 든다.	⓪	①	②	③
s20	심장이 크게 뛰거나 빠르게 뛴다.	⓪	①	②	③
s21	떨린다.	⓪	①	②	③
s22	긴장되거나 예민해진다.	⓪	①	②	③
s23	두통이 있다.	⓪	①	②	③
s24	극도의 공포나 불안을 경험한다.	⓪	①	②	③
s25	안절부절 못하고 가만히 앉아 있지도 못한다.	⓪	①	②	③

※ [q02] 다음은 귀하께서 평소 자신에 대해 어떻게 느끼고 생각하고 있는지에 대한 질문입니다. 각 문항을 읽고 자신의 상태와 가장 가까운 번호에 ○표 하십시오.

번호	내 용	전혀 그렇지 않다	그렇지 않다	그렇다	전적으로 그렇다
s1	나는 계획대로 일을 수행할 수 있다.	①	②	③	④
s2	나는 일을 해야 할 때 바로 일을 시작하지 못하는 문제점이 있다.	①	②	③	④
s3	나는 어떤 일을 처음에 잘못하더라도 해낼 때 까지 해본다.	①	②	③	④
s4	나는 중요한 목표를 설정하면 성취할 수 있다.	①	②	③	④
s5	나는 어떤 일을 끝마치기도 전에 포기한다.	①	②	③	④
s6	나는 어려운 일에 부딪히는 것을 피한다.	①	②	③	④
s7	나는 어떤 일이 너무 복잡해 보이면 해 볼 시도조차 안한다.	①	②	③	④
s8	나는 별로 내키지 않은 어떤 일을 할 때도 그것을 끝마칠 때까지 반드시 한다.	①	②	③	④
s9	나는 뭔가 할 일이 있을 때 바로 그 일을 시작한다.	①	②	③	④
s10	새로운 어떤 일을 배우려고 시도할 때 처음에 성공할 것 같지 않으면 바로 포기한다.	①	②	③	④
s11	예상치 못한 문제가 일어나면 나는 잘 대처할 수 있다.	①	②	③	④
s12	나는 어떤 새로운 일이 너무 어려우면 배우려고 시도하지 않는다.	①	②	③	④
s13	실패는 나로 하여금 더 열심히 노력하도록 만들 뿐이다.	①	②	③	④
s14	나는 어떤 일을 할 수 있는지 내 능력에 불안감을 느낄 때가 있다.	①	②	③	④
s15	나는 자신감이 있다.	①	②	③	④
s16	나는 쉽게 포기한다.	①	②	③	④
s17	나는 인생에 부딪히는 거의 모든 문제들을 다룰 능력이 없는 것 같다.	①	②	③	④
s18	새로운 친구를 사귀는 일은 내게 너무 어려운 일이다.	①	②	③	④
s19	나는 어떤 사람이 보고 싶으면 그 사람이 와주기를 기다리는 대신 내가 먼저 간다.	①	②	③	④
s20	내가 관심을 가지는 어떤 사람이 사귀기 어려운 사람이라면 나는 사귀는 것을 금방 포기한다.	①	②	③	④
s21	첫 눈에 호감이 가지 않는 사람이라 해도 나는 그 사람과 사귀는 것을 쉽게 포기하지 않는다.	①	②	③	④
s22	나는 사회적(사교적) 모임에서 내 자신을 어찌해야 좋을지 모르겠다.	①	②	③	④
s23	나는 지금의 내 친구들을 내 사귐성 덕분에 사귀었다.	①	②	③	④

※ [q03] 다음은 귀하에 대한 생각을 질문한 것입니다. 자신의 생각을 잘 나타낸 곳에 ○표 하십시오.

번호	내 용	전혀 그렇지 않다	대체로 그렇지 않다	대체로 그렇다	항상 그렇다
s1	나는 내가 다른 사람들처럼 가치있는 사람이라고 생각한다.	①	②	③	④
s2	나는 내가 좋은 인품을 가졌다고 생각한다.	①	②	③	④
s3	나는 내가 전반적으로 실패한 사람이라는 느낌이 든다.	①	②	③	④
s4	나는 대부분의 다른 사람들과 같이 일을 잘 할 수가 있다.	①	②	③	④
s5	나는 스스로 자랑할 것이 별로 없다.	①	②	③	④
s6	나는 내 자신에 대하여 긍정적인 태도를 가지고 있다.	①	②	③	④
s7	나는 내 자신에 대하여 전반적으로 만족한다.	①	②	③	④
s8	나는 내 자신을 좀 더 존경할 수 있으면 좋겠다.	①	②	③	④
s9	나는 가끔 내 자신이 쓸모없는 사람이라는 느낌이 든다.	①	②	③	④
s10	나는 때때로 내가 좋지 않은 사람이라고 생각한다.	①	②	③	④

※ [q04] 다음은 귀하의 어릴 적 경험(16세 이전)에 대한 질문입니다. 자신이 아래와 같은 일을 16세 이전에 경험하였다면 '예'에, 경험하지 않았다면 '아니오' 에○표 하십시오. (예->1, 아니오->2)

번호	경험 내용	예	아니오
s1	부모 중 한 사람이라도 사망하였다.	예	아니오
s2	부모나 가족으로부터 심한 신체적 학대를 받았다.	예	아니오
s3	폭언과 구박과 같은 정신적 학대를 받았다.	예	아니오
s4	성적 추행이나 학대를 받았다.	예	아니오
s5	부모가 나를 버렸거나, 다른 곳으로 멀리 보냈다.	예	아니오
s6	부모가 이혼이나 별거를 하였다.	예	아니오
s7	부모 중 한사람이라도 음주문제로 일상, 가정생활, 직업활동에 지장이 있었다.	예	아니오
s8	부모 중 한사람이라도 정신질환으로 일상, 가정 또는 직장생활에 지장이 있었다.	예	아니오
s9	가족이나 가까운 친구가 죽거나 죽을 뻔한 것을 직접 보았다.	예	아니오
s10	심한 질병이나 사고로 생명의 위협이 있었거나 장기간 앓아 누웠다.	예	아니오
s11	질병이나 사고로 영구적인 또는 상당기간 지속된 장애를 가지게 되었다.	예	아니오
s12	가족이나 친구가 자살로 죽거나 자살을 시도하였다.	예	아니오

※ [q05~q06] 다음은 <u>일반적으로 북한이주민들이 북한에서 혹은 탈북과정에서 경험했을 수도 있는</u> 일입니다. 자신이 아래와 같은 일을 직접 <u>경험하셨으면</u> '예'에, 경험하지 않았으면 '아니오'에 ○표 하십시오. (예->1, 아니오->2)

번호	문 항	북한 [q05]		탈북 후 부터 한국입국 전까지 [q06]	
		예	아니오	예	아니오
s1	추위나 식량부족으로 인해 생명의 위협을 받았다.	예	아니오	예	아니오
s2	총격이나 추격을 받았다.	예	아니오	예	아니오
s3	아주 심하게 매를 맞았다.	예	아니오	예	아니오
s4	수용소, 교화소나 감옥에 간 적이 있다.	예	아니오	예	아니오
s5	나의 출신성분이나 정치적 과오로 인해 사상성을 의심받았다.	예	아니오	예	아니오
s6	가족이나 친지의 정치적 과오로 인해 내가 처벌을 받았다.	예	아니오	예	아니오
s7	가족과 생이별을 하게 되었다.	예	아니오	예	아니오
s8	가족이나 가까운 친지가 식량을 구하기 위해 떠난 후 소식이 끊어졌다.	예	아니오	예	아니오
s9	심각한 질병으로 죽을 고비를 겪었거나 장애가 생겼다.	예	아니오	예	아니오
s10	가족, 친지, 가까운 이웃 중에 굶어 죽는 것을 목격하거나 소식을 들었다.	예	아니오	예	아니오
s11	아는 사람이 공개처형 당하는 것을 목격했다.	예	아니오	예	아니오
s12	고문을 당했다.	예	아니오	예	아니오
s13	가족이나 가까운 친지 중 자살을 하거나 갑작스레 죽게 된 사람이 있다.	예	아니오	예	아니오
s14	홍수나 산불 등 자연피해(재해)를 당했다.	예	아니오	예	아니오
s15	심한 성적 모욕이나 강간을 당했다.	예	아니오	예	아니오
s16	원치 않는 강제 결혼을 했거나 인신매매를 당했다.	예	아니오	예	아니오
s17	나를 도와주기로 약속한 사람에게 예상치 못한 배신을 당했다	예	아니오	예	아니오
s18	체포나 강제 북송된 경험이 있었거나, 거의 그럴 뻔한 위험에 처했다.			예	아니오

※ [q07] <u>한국 입국 후 처음 정착했던 시기와 현재를 비교 할 때</u> 자신의 일상생활에서의 변화가 어떠한지에 대한 질문들입니다. 자신의 상황을 가장 잘 나타내는 부분에 ○표 하십시오.

번호	문 항	그렇지 않다	조금 그렇다	보통 그렇다	매우 그렇다
s1	나는 다른 사람들을 더욱 배려할 수 있게 되었다.	①	②	③	④
s2	나 스스로 결정하는 것에 대해 훨씬 자유로워짐을 느꼈다.	①	②	③	④
s3	내 인생에도 다른 사람과 나눌만한 가치있는 것이 있다는 것을 알게 되었다.	①	②	③	④
s4	내가 원하는 내 자신의 모습을 찾아가게 되었다.	①	②	③	④
s5	나는 어려움을 포기하지 않고 극복할 수 있다는 자신감이 생겼다.	①	②	③	④
s6	나는 인생의 의미를 더 많이 찾을 수 있게 되었다.	①	②	③	④
s7	나는 다른 사람들에게 어떻게 다가가서 도움을 줘야하는지 알게 되었다.	①	②	③	④
s8	나는 더 자신감 있는 사람이 되었다.	①	②	③	④
s9	다른 사람들이 나에게 이야기 할 때 마음을 기울여 들어줄 수 있게 되었다.	①	②	③	④
s10	새로운 정보나 생각들을 받아들이기 쉬워졌다.	①	②	③	④
s11	나는 다른 사람들과 더 솔직하게 대화할 수 있게 되었다.	①	②	③	④
s12	나도 다른 사람들에게 좋은 영향을 줄 수 있는 사람이 되고 싶다는 확신이 들었다.	①	②	③	④
s13	어려움이 있을 때 다른 사람들에게 도움을 청해도 괜찮다는 것을 알게 되었다.	①	②	③	④
s14	내 자신의 개인적인 권리를 말 할 수 있게 되었다.	①	②	③	④
s15	내가 생각했던 것 보다 내게 관심을 가져 주는 사람들이 많아졌다.	①	②	③	④

5. 음 주 (c5)

※ 다음은 귀하의 음주에 관한 질문입니다. 자신의 음주 태도를 잘 나타낸 번호를 □안에 써 주십시오.

q01) 2008년 1년간 평균적으로 귀하는 술을 얼마나 자주 마셨습니까? □

⓪ 전혀 마시지 않는다 ▶ **C 설문지를 마치셨습니다.**
① 월 1회 미만
② 월 2-4회
③ 주 2-3회
④ 주 4회 이상

q02) 술을 마시면 보통 몇 잔을 마십니까?-- □

⓪ 1-2잔 ① 3-4잔 ② 5-6잔 ③ 7-9잔 ④ 10잔 이상

q03) 한 번에 소주 1병 또는 맥주 4병 이상 마시는 경우는 얼마나 자주 있습니까?---------------------------- □

⓪ 없다 ① 월 1회 미만 ② 월 1회 ③ 주 1회 ④ 거의 매일

q04) 2008년 1년간 술을 마시기 시작하여 멈출 수 없었던 때가 얼마나 자주 있었습니까?------------------- □

⓪ 전혀 없다 ① 한달에 한번 ② 한달에 2-3회 ③ 일주일에 한번 ④ 거의 매일

q05) 2008년 1년간 평소 같으면 할 수 있었던 일을 음주 때문에 못한 적이 얼마나 자주 있었습니까?----- □

⓪ 전혀 없다 ① 월 1회 미만 ② 월 1회 ③ 주 1회 ④ 거의 매일

q06) 2008년 1년간 술을 많이 마신 다음 날, 일을 나가기 위해 해장술이 필요했던 적은 얼마나 자주 있었습니까?---- □

⓪ 전혀 없다 ① 월 1회 미만 ② 월 1회 ③ 주 1회 ④ 거의 매일

q07) 2008년 1년간 음주 후에 죄책감이 들거나 후회를 한 적이 얼마나 자주 있었습니까?------------------- □

⓪ 전혀 없다 ① 월 1회 미만 ② 월 1회 ③ 주 1회 ④ 거의 매일

q08) 2008년 1년간 음주 때문에 전날 밤 일이 기억나지 않았던 일이 얼마나 자주 있었습니까?------------ □

⓪ 전혀 없다 ① 월 1회 미만 ② 월 1회 ③ 주 1회 ④ 거의 매일

q09) 2008년 1년간 음주로 인해 자신이나 다른 사람이 다친 적이 있었습니까?-------------------------------- □

⓪ 전혀 없다 ① 월 1회 미만 ② 월 1회 ③ 주 1회 ④ 거의 매일

q10) 2008년 1년간 친척이나 친구, 또는 의사가 귀하의 술 마시는 것에 대해 걱정하거나 술 끊기를 권유한 적이 있었습니까?-- □

⓪ 전혀 없다 ① 월 1회 미만 ② 월 1회 ③ 주 1회 ④ 거의 매일
